# Chromosome Painting Reveals Asynaptic Full Alignment of Homologs and HIM-8–Dependent Remodeling of *X* Chromosome Territories during *Caenorhabditis elegans* Meiosis

**DOI:** 10.1371/journal.pgen.1002231

**Published:** 2011-08-18

**Authors:** Kentaro Nabeshima, Susanna Mlynarczyk-Evans, Anne M. Villeneuve

**Affiliations:** 1 Department of Cell and Developmental Biology, University of Michigan Medical School, Ann Arbor Michigan, United States of America; 2 Departments of Developmental Biology and Genetics, Stanford University School of Medicine, Stanford, California, United States of America; Stowers Institute for Medical Research, United States of America

## Abstract

During early meiotic prophase, a nucleus-wide reorganization leads to sorting of chromosomes into homologous pairs and to establishing associations between homologous chromosomes along their entire lengths. Here, we investigate global features of chromosome organization during this process, using a chromosome painting method in whole-mount *Caenorhabditis elegans* gonads that enables visualization of whole chromosomes along their entire lengths in the context of preserved 3D nuclear architecture. First, we show that neither spatial proximity of premeiotic chromosome territories nor chromosome-specific timing is a major factor driving homolog pairing. Second, we show that synaptonemal complex-independent associations can support full lengthwise juxtaposition of homologous chromosomes. Third, we reveal a prominent elongation of chromosome territories during meiotic prophase that initiates prior to homolog association and alignment. Mutant analysis indicates that chromosome movement mediated by association of chromosome pairing centers (PCs) with mobile patches of the nuclear envelope (NE)–spanning SUN-1/ZYG-12 protein complexes is not the primary driver of territory elongation. Moreover, we identify new roles for the *X* chromosome PC (*X*-PC) and *X*-PC binding protein HIM-8 in promoting elongation of *X* chromosome territories, separable from their role(s) in mediating local stabilization of pairing and association of *X* chromosomes with mobile SUN-1/ZYG-12 patches. Further, we present evidence that HIM-8 functions both at and outside of PCs to mediate chromosome territory elongation. These and other data support a model in which synapsis-independent elongation of chromosome territories, driven by PC binding proteins, enables lengthwise juxtaposition of chromosomes, thereby facilitating assessment of their suitability as potential pairing partners.

## Author Summary

Successful sexual reproduction relies on the ability of germ cells to faithfully segregate homologous chromosomes in meiosis, which requires accurate sorting of chromosomes into homologous pairs and alignment of homologs along their entire lengths. The mechanisms underlying homolog sorting and alignment are not well understood, partly because of a scarcity of studies investigating homolog alignment at the level of whole chromosomes. This study provides a global view of the organization of chromosome territories during early meiotic prophase in the nematode *Caenorhabditis elegans*. We applied chromosome painting to visualize the entire lengths of chromosomes. Our study provides several conceptual advances. First, our study excluded several possible mechanisms as primary drivers of chromosome sorting. Second, our analysis has revealed both a robust capacity for full-lengthwise alignment between homologous chromosomes prior to the stabilization of pairing by the synaptonemal complex as well as a dramatic elongation of chromosome territories that could enable this alignment. We also identified a factor required for the elongation of chromosome territories. Elongation of chromosome territories could enable lengthwise juxtaposition of chromosomes, thereby facilitating assessment of their suitability as potential pairing partners by promoting utilization of information about chromosome identity that is distributed along the length of a chromosome.

## Introduction

The success of sexual reproduction relies on the ability of diploid germ cells to generate haploid gametes through the specialized cell division program of meiosis. Haploidization relies on the faithful segregation of chromosomes from their homologous partners, which in turn relies on an ability to sort chromosomes into homologous pairs and establish temporary associations between them. It is now well established that both recombinational interactions at the DNA level and assembly of a meiosis-specific proteinaceous structure known as the synaptonemal complex (SC) play roles in stabilizing associations between homologous chromosomes. However, how homologs become colocalized and how initial recognition is accomplished to establish these associations remains poorly understood.

Substantial progress has been made recently in illuminating a conserved mechanism that mediates chromosome movements that likely contribute to the chromosome sorting process in diverse organisms (for reviews, see [1]–[3]). The common feature of these large-scale spatial reorganization mechanisms involves tethering of one or two specified site(s) on a chromosome to conserved nuclear envelope (NE)- spanning protein complexes, thereby coupling the chromosomes to the cytoskeletal motility apparatus that can transmit forces to drive chromosome movement. Such forces and movements have been proposed to enhance the efficiency of chromosome sorting both by providing opportunities for homology assessment and by destabilizing interactions between incorrect partners. However, our understanding remains limited regarding how localized chromosome tethering sites might mediate recognition and bring about colocalization along the entire lengths of chromosomes.

One reason for this limitation is that many studies of early prophase chromosome reorganization have focused on assessing the behavior of a few specified individual chromosomal loci. While such approaches have been highly fruitful, they do not provide information regarding the spatial and morphological organization of whole chromosomes within intact nuclei prior to and during the pairing process. Intermediate events in the pairing process are particularly difficult to investigate using single-locus visualization methods, as only two pairing states (paired or unpaired) can be captured at each locus. Thus in order to comprehensively study the pairing process, including intermediate states, it is necessary to study pairing in the context of whole chromosomes. One cytological approach that has been applied to address this problem is the use of “chromosome paints”, *i.e.*, fluorescence *in situ* hybridization (FISH) probes that allow visualization of whole chromosomes or large chromosome segments. Several previous studies have applied whole chromosome paints to investigate meiotic pairing, *e.g.* to visualize endogenous chromosomes in human spermatocytes [4]–[6] or “alien” chromosomes in hybrid plants [7]–[9]. Such studies have indeed succeeded in revealing aspects that would be difficult to demonstrate without whole chromosome visualization, such as intermediate pairing states along the chromosome before synapsis [10] and a correlation between pairing activity and morphological changes in heterochromatin in plants [9], [11]–[13]. However, technical challenges such as limited availability of material (*e.g.*, human) or difficulty visualizing native chromosome complements rather than exogenously-derived chromosomes (*e.g.*, plants) have hindered widespread adoption of the paint approach.

In the current work, we have applied a multi-color whole-chromosome painting approach to visualize spatial and morphological reorganization of chromosome territories during meiotic prophase in the nematode *Caenorhabditis elegans*, which has several features that make it especially amenable to reaping the benefits of the painting approach. Germ cells comprise more than half of the cells in the adult hermaphrodite and are organized in a spatial-temporal gradient along the longitudinal axis of the gonad, facilitating analysis of large numbers of nuclei undergoing the meiotic pairing process [14], [15]. Further, the germ line is organized as an optically clear single-layer epithelial tube, enabling visualization of chromosome territories in the context of well-preserved 3D nuclear architecture in whole mount gonads. Finally, these cytological advantages can be exploited in combination with a rich history of genetic analysis of meiosis that has identified both *cis-* and *trans-*acting factors required to establish and maintain homolog pairing (Reviewed in [16]).

A central player driving the chromosome sorting process in *C. elegans* is the meiotic pairing center (PC), a *cis*-acting domain located near one end of each chromosome [17], [18]. PCs have been demonstrated to play roles both in promoting local stabilization of pairing and in promoting SC assembly; these dual roles of PCs together ensure that synapsis occurs specifically between homologous chromosomes [19], [20]. PC function depends on members of a family of Zn finger DNA binding proteins (HIM-8 and ZIM-1, -2, -3), each of which becomes concentrated at the PCs of a specific subset of chromosomes [21]–[23]. Recent work has shown that PCs are the sites at which *C. elegans* chromosomes associate with conserved NE-spanning protein complexes (comprised of the SUN-1 and ZYG-12 proteins) that mediate tethering of chromosomes to the cytoskeletal motility apparatus to enable chromosome movement during meiotic prophase [24]–[26]. Further, PCs have been proposed to participate in checkpoint-like mechanisms that function, in a manner analogous to the spindle-assembly checkpoint, to prevent licensing of SC assembly until successful homologous associations have been achieved [25], [27], [28]. While PCs represent a key focal point for activities that drive chromosome movements, mediate associations between prospective pairing partners, and couple SC assembly to homology verification, however, it is not clear how the known functions and properties of PCs and PC binding proteins might contribute to homolog recognition *per se*. Further, our current understanding of PCs does not explain how chromosomal regions outside of the PCs might contribute to homology recognition.

In the current work, we fill a major gap in our knowledge regarding the spatial and morphological organization of chromosomes within *C. elegans* germ cell nuclei prior to meiotic entry and during the process of homolog pairing. Visualization of whole chromosome territories has allowed us to exclude several possible mechanisms as primary drivers of chromosome sorting. Further, our analysis has revealed both a robust capacity for synapsis-independent full-lengthwise alignment between homologous chromosomes as well as a dramatic longitudinal extension of chromosome territories that could enable this alignment. Moreover, we demonstrate unanticipated roles for the *X* chromosome PC and PC binding protein HIM-8 in promoting territory elongation. These and other observations together paint a picture in which longitudinal restructuring of chromosome territories collaborates with other functions of meiotic pairing centers to bring about efficient, stable and productive interactions between homologous chromosomes, ultimately leading to the production of haploid gametes.

## Results

### Visualizing chromosome territories with chromosome paints in whole-mount *C. elegans* gonads

**Figure 1:**
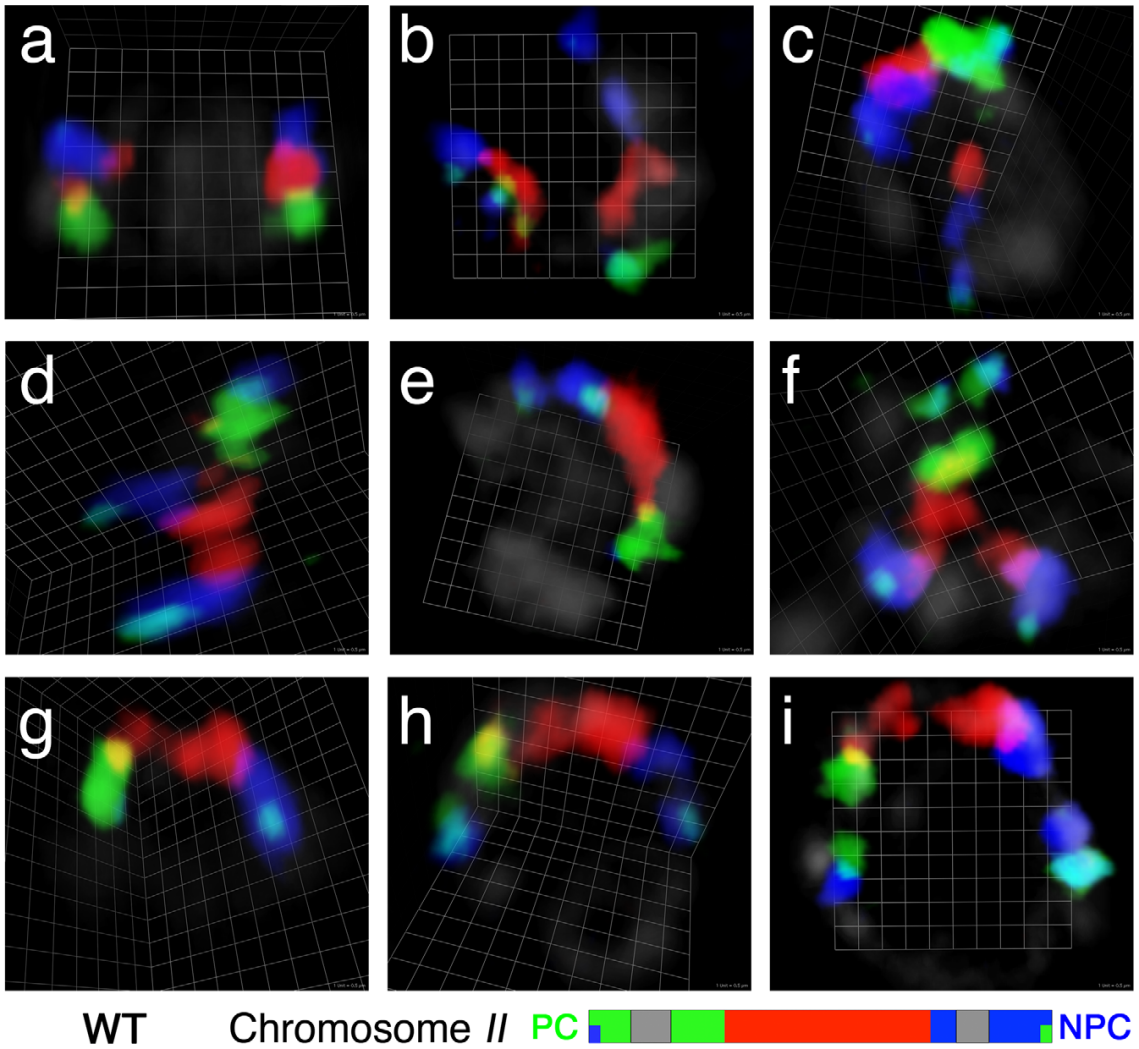
Visualization of chromosome *II* territories using multicolor chromosome paints. Each panel shows a three-dimensional rendered image of the chromosome *II* pair in a single wild-type nucleus from a whole-mount gonad, reconstructed from optical sectioning confocal microscopy; the chromosomes are visualized using a multicolor chromosome paint FISH probe, which is depicted schematically below. Three different fluorescent dyes are assigned to each of three segments of chromosome *II*: Alexa-488 to the left segment (green), Alexa-555 to the central segment (red) and Alexa-647 to the right segment (blue). (Details of the labeling scheme are in Table S1). The left and right ends of the chromosomes are double-labeled with a second fluorescent dye (Alexa-647 and Alexa-488, respectively), and appear light blue in color as a result of the colocalization of the two dyes. In this and other figures, gray segments in the schematic indicate regions of the chromosome that were not represented in the probe; PC indicates the pairing center end and NPC represents the non-pairing center end of the relevant chromosome. In the images, DAPI counter stain is shown in light gray. (a) A nucleus in the pre-meiotic zone, in which the two homologous chromosomes are seen in compact territories. (b–g) Nuclei from the transition zone (TZ), the region of the germ line where chromosome territories become longitudinally extended and pairing is established. Within the TZ, homologs can be: (b) not aligned; (c–f) partially aligned; or (g) fully aligned. (h and i) Nuclei from the pachytene zone, in which fully aligned homologous chromosomes are seen in a single chromosome territory progressively extending during pachytene progression. Scale is shown by the square grid in the background of each panel, with 0.5 µm as the length of each side of the unit square.

During meiotic prophase, chromosomes are spatially reorganized within the nucleus to establish productive associations between homologous chromosomes. In order to study this reorganization process, we used a chromosome painting approach to visualize complete chromosomal territories in the context of preserved 3D nuclear architecture in whole mount gonads of *C. elegans*. This method provides a means to trace the full length of a chromosome, making it particularly useful for analyzing the spatial organization of chromosomes in premeiotic nuclei and during intermediate steps in chromosome alignment. Figure 1 summarizes representative observations from an experiment in which chromosome *II* was visualized with a paint probe generated from YAC clones tiled along the length of the chromosome (see Materials and Methods). For this probe, three different fluorescent dyes were assigned to three distinct segments of the chromosome; in addition, the left- and right-most YACs were double-labeled with a second fluorophore in order to clearly locate chromosome ends.

We exploited the stereotyped spatio-temporal organization of nuclei within the *C. elegans* gonad and the appearance of DAPI-stained chromatin to identify premeiotic nuclei and nuclei at different stages of meiotic prophase. Several basic observations are introduced briefly here and will be expanded upon in subsequent sections: 1) In premeiotic nuclei (located in the distal region of the germ line), the chromosome *II* paint probe typically labels two relatively compact ovoid territories, often widely separated within the nucleus (Figure 1a). 2) As nuclei move proximally in the gonad, they enter meiotic prophase and initiate homolog pairing in a region known as the transition zone. The transition zone contains nuclei in the leptotene and zygotene stages of meiotic prophase, and is characterized by an asymmetric clustering of chromosomes within the nucleus that reflects active chromosome movement driven by connections of chromosomes to the cytoskeletal motility apparatus [25], [26]. Nuclei within the transition zone frequently exhibit highly elongated chromosome territories; such elongated chromosome territories may be unassociated (Figure 1b), partially aligned (Figure 1c–1f) or completely aligned along their entire lengths (Figure 1g). At this and later stages, chromosome paint signals sometimes exhibit a discontinuous “beads-on-a-string” appearance, even for chromosome regions whose sequence is fully represented by YAC clones in the paint probe. 3) Nuclei exit the transition zone upon entry into the pachytene stage, during which homologous chromosomes are fully aligned and stably associated with their homologous partners via the synaptonemal complex (SC), and chromosomes are redispersed around the nuclear periphery. During the pachytene stage, chromosome paints reveal a single elongated chromosome territory reflecting complete alignment and association of homologous chromosomes along their entire lengths (Figure 1h, 1i).

### Preferential proximity between homologous territories is established predominantly after meiotic entry

**Figure 2:**
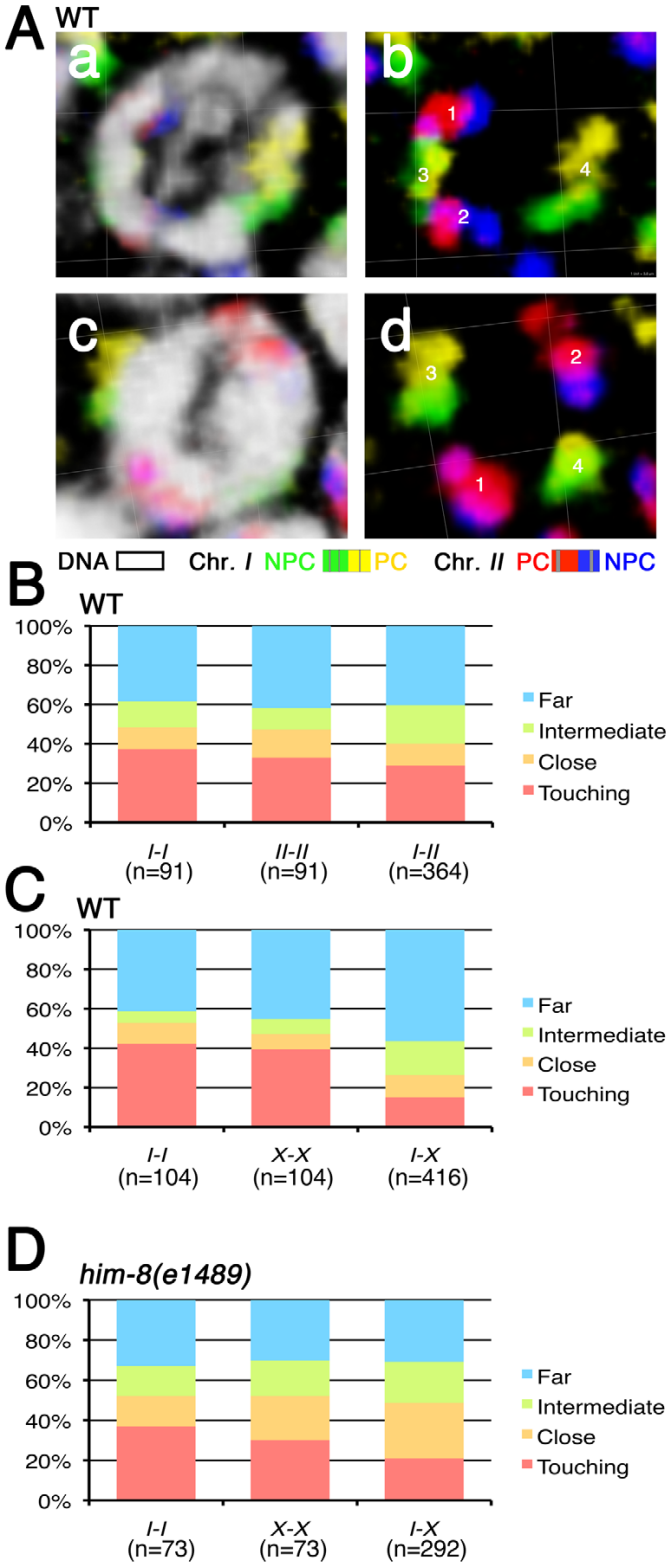
Spatial organization of premeiotic chromosome territories. (A) Three-dimensional rendered paint images of chromosomes *I* and *II* in individual wild-type nuclei in the pre-meiotic zone. Chromosome *I* is painted with Alexa-488 (green) on the left half and Alexa-532 (yellow) on the right half. Chromosome *II* is painted with Alexa-594 (red) on the left half and Alexa-647 (blue) on the right half. DAPI counter staining is shown in white in panels a and c. The spatial relationships between each pair of chromosome territories in each nucleus were categorized based on the observed separation between the two territories in the 3D reconstruction. In the nucleus in panel b, the distance between territories 1 and 2 was scored as “intermediate” (D<d≤2D, where d = the shortest distance between the edges of two territories and D = the average width of a territory); distances between 1 and 3 and between 2 and 3 were both scored as “touching”; the distance between territories 3 and 4 was scored as “far” (d>2D). In panel d, the distance between territories 1 and 3 was scored as “close” (d<D, but the territories are not touching). Scale is shown by the square grid in the background of each panel, with 3.8 µm as the length of each side of the unit square. (B–D) Quantification of the spatial organization of premeiotic chromosome territories. (B) Stacked bar graphs indicate the fraction of distances between pairs of homologous (*I-I* and *II-II*) or heterologous (*I-II*) territories in wild type worms in each of the four spatial-relationship categories (Far, Intermediate, Close and Touching) described above. A total of 91 nuclei from the pre-meiotic regions of three germ lines was analyzed. (C) Stacked bar graphs showing quantification of the spatial organization of chromosome territories for chromosome *I* and the *X* chromosome in wild type worms. 104 nuclei from three germ lines were analyzed. (D) Stacked bar graphs showing quantification of the spatial organization of chromosome territories for chromosome *I* and the *X* chromosome in *him-8*(*e1489*) mutant worms. 73 nuclei from three germ lines were analyzed.

In principle, a prior non-random arrangement of chromosomes within the nucleus could potentially contribute mechanistically to the process of homolog pairing during meiosis. It was previously shown that homologous chromosomes are not aligned prior to meiotic entry in *C. elegans*, as pairing between homologous loci in premeiotic nuclei is rarely observed by conventional locus-specific FISH (*e.g.* [19], [29], [30]). However, it had not been addressed whether territories of homologous chromosomes might exhibit preferential proximity compared to non-homologous territories. Therefore, we compared the spatial relationships between homologous and non-homologous territories by simultaneously visualizing two pairs of chromosomes in a subset of the pre-meiotic zone (1–15 cell diameters from the distal tip) that does not include pre-meiotic S phase. These experiments used four fluorescent dyes (two assigned to each chromosome) to identify each chromosome and to distinguish their left and right halves. Spatial relationships between chromosome territories were examined in 3D reconstructed images (Figure 2A). We defined four categories of spatial proximity for this analysis. If we observed any overlap or association between two territories, territories were scored as “touching”. Other spatial relationships between two territories were classified into three categories based on visual inspection of 3D-rendered images. We approximated the distances between the closest edges of the territories (d) using the width of chromosome territory as a scale unit (D), since these territories are highly uniform in their width (0.67 µm±SD 0.05 µm, n = 16); distances were classified as: close (0<d≤D), intermediate (D<d≤2D) or far (2D≤d).

Several features of premeiotic nuclear organization were revealed by simultaneous analysis of chromosome *I* and *II* territories (Figure 2B). First, homologous chromosome territories were frequently widely spaced within the nucleus, with more than half of the distances in the intermediate and far categories. Second, both homologous pairs (*I-I* and *II-II*) exhibited similar distributions among the different spatial relationship categories. Third, heterologous pairs of chromosome territories (*I*-*II*) did not exhibit a significantly different distribution among the spatial categories compared with homologous (*I-I* or *II-II*) pairs (p = 0.34 and p = 0.26). This indicates that these chromosomes are not “presorted” in most of the pre-meiotic stage and suggests that premeiotic nuclear organization is not the primary factor driving meiotic homolog pairing.

Simultaneous analysis of chromosome *I* and *X* chromosome territories revealed both similarities and differences (Figure 2C). On one hand, the *X* chromosomes were no more or less likely than chromosomes *I* or *II* to be in close proximity to or associated with their homologous partners, as the distributions among the spatial categories did not exhibit any significant differences (p = 0.79, p = 0.36). This reinforces the view that no chromosome pair is in a preferentially pre-associated state in the pre-meiotic stage. On the other hand, however, heterologous pairs of chromosome territories (*I*-*X*) did exhibit a significantly different distribution among the spatial categories when compared with homologous (*I-I* or *X-X*) pairs (p<0.0001), reflecting a tendency of *I* and *X* to be separated by larger distances. This observation raises the possibility that the *X* chromosomes, while no more likely than autosomes to be closely associated with each other, may nevertheless be spatially segregated from autosomes in pre-meiotic nuclei. Such a feature could help explain the higher proficiency of *X* chromosome pairing under some conditions where autosomal pairing is severely abrogated [27], [28], [31]–[34].

One possible reason why the *X* chromosomes might be spatially segregated from the autosomes premeiotically is that *X* chromosome PCs exhibit strong association with the HIM-8 protein and the nuclear envelope in premeiotic nuclei, whereas the ZIM proteins do not show strong association with the autosomal PCs until meiotic entry [21], [22]. We tested whether HIM-8 might be responsible for X-A spatial segregation by examining the spatial organization of pre-meiotic territories in the *him-8*(*e1489*) mutant, which lacks detectable HIM-8 protein. We found that the tendency for the *X* chromosome to be spatially segregated from chromosome *I* was diminished in the *him-8(e1489)* mutant (Figure 2D), as the distribution among spatial categories for heterologous pairs (*I*-*X*) did not differ significantly from the distribution observed for *X*-*X* pairs (p = 0.21) and exhibited only a modest difference from that observed for *I*-*I* pairs (p = 0.03). Thus, we conclude that association of the *X* chromosomes with the HIM-8 protein and/or the nuclear envelope does indeed contribute to the spatial segregation of *X* chromosomes from autosomes in premeiotic nuclei.

### Chromosomes do not exhibit a temporal hierarchy for homolog alignment

**Figure 3:**
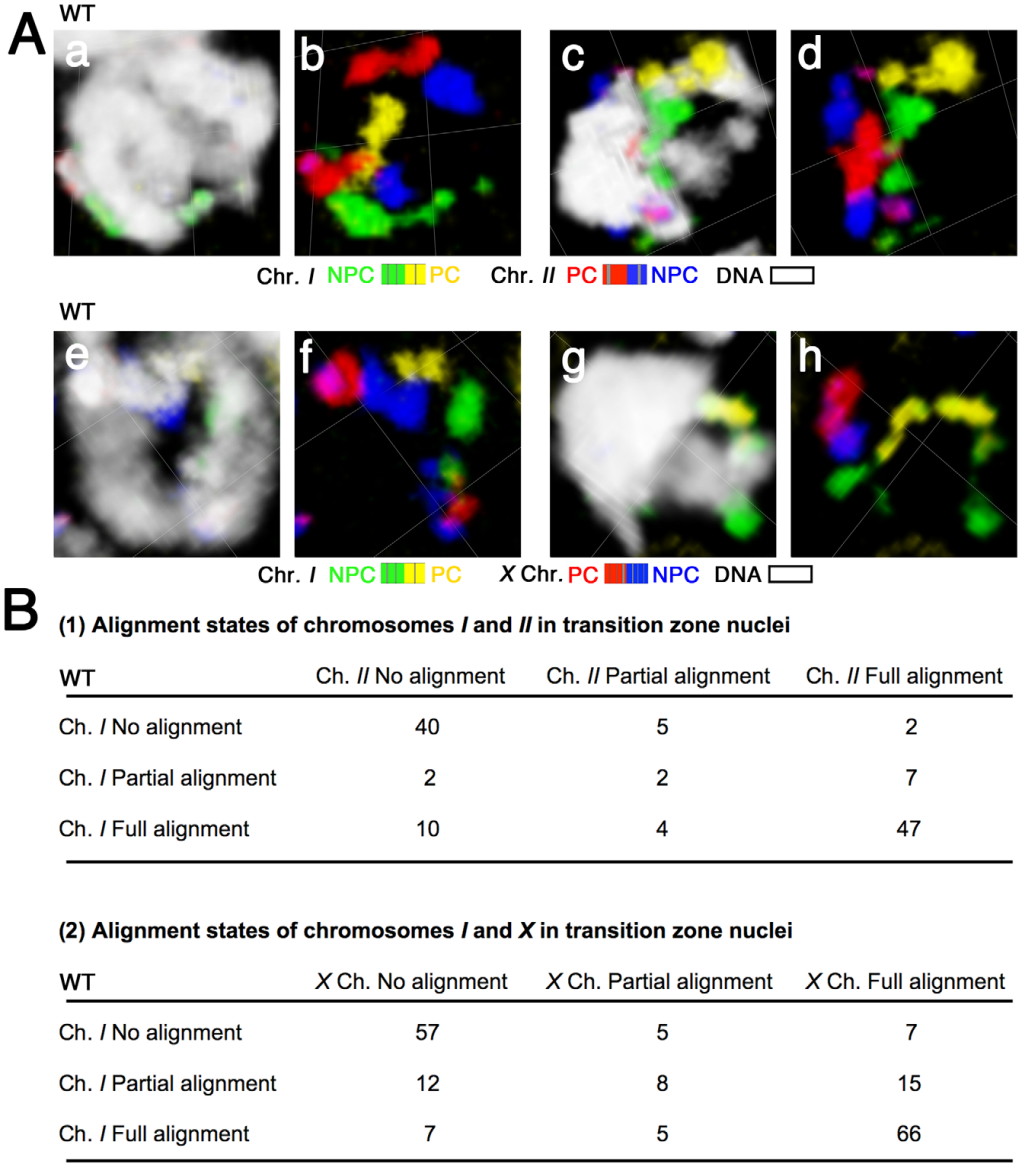
Chromosomes do not exhibit a temporal hierarchy for homolog alignment in the transition zone. (A) Three-dimensional rendered paint images of chromosomes *I* and *II* (a–d), and chromosomes *I* and *X* (e–h) in individual nuclei from the transition zone. Chromosomes *I* and *II* are painted as in Figure 2A. The *X* chromosome is painted with Alexa-594 (red) on the left half and Alexa-647 (blue) on the right half. DAPI counter staining is shown in white. (a, b) a fully aligned chromosome *I* pair is seen as a single territory, whereas two distinct chromosome *II* territories indicate a lack of association between the chromosome *II* homologs. (c, d) full alignment of chromosome *I* and partial alignment (in the red segment) of chromosome *II*. (e, f) fully aligned chromosome *I* and no association between the *X* chromosomes. (g, h) full alignment of the *X* chromosomes and partial alignment (in the yellow segment) of chromosome *I*. Scale is shown by the square grid in the background of each panel, with 3.8 µm as the length of each side of the unit square. (B) Matrices presenting quantitative analysis of chromosome alignment states in transition zone nuclei. Nuclei were classified into nine categories based on the alignment states of chromosomes *I* and *II* (Part 1, n = 119 nuclei) or chromosomes *I* and *X* (Part 2, n = 182 nuclei).

The same sets of paints (4-color, 2-chromosome) were used to examine chromosome organization in transition zone nuclei, where homolog pairing is established. This analysis enabled us to investigate the possibility that temporal heterogeneity in chromosome organization or behavior might play a role in chromosome sorting during the pairing phase of meiotic prophase. Specifically, for each transition zone nucleus we assessed whether each homologous chromosome pair was unaligned, partially aligned or fully aligned (Figure 3). In the majority of transition zone nuclei, we found that either both pairs of chromosomes were unaligned or both pairs were completely aligned, presumably reflecting nuclei that had either not yet begun or had already completed the homolog alignment process. Thus, to address the issue of relative timing, we specifically considered the subset of nuclei that were in the process of achieving homolog alignment, *i.e.*, those nuclei in which either 1) both chromosome pairs exhibited partial alignment or 2) the two chromosome pairs exhibited disparate alignment states (Figure 3A). Importantly, there was no pattern among these nuclei that would indicate a specific temporal hierarchy in the order of chromosome pairing. We observed nuclei in which chromosome *I* was partially or fully aligned and chromosome *II* was unaligned, and we likewise found nuclei in which chromosome *II* was partially or fully aligned and chromosome *I* was unaligned. Similar results were obtained for *I* and *X*. Thus, these data demonstrate that all of the chromosomes initiate and complete homologous alignment in a very similar time window. Moreover, these experiments rule out the possibility that different chromosomes within the nucleus might pair in a reproducible temporal order.

### Homologous chromosomes can achieve full alignment in the absence of synapsis

During wild-type *C. elegans* meiosis, successful pairing of homologous chromosomes is quickly stabilized by assembly of the SC, making it difficult to determine the extent to which full alignment between homologs can occur in the absence of synapsis. Mutants lacking the SC central region proteins (*syp* mutants) provide an opportunity to address this issue, as such mutants not only lack synapsis but also prolong the period of chromosome clustering and chromosome mobility associated with the onset of homolog pairing. Previous work using locus-specific FISH probes had shown that *syp* mutants achieve substantial levels of pairing at the pairing center (PC) ends of their chromosomes, revealing a role for PCs in promoting local synapsis-independent stabilization of pairing [19]. A subset of chromosomes in this prior analysis displayed paired FISH signals at both PC and non-PC ends, consistent with the possibility of full alignment, but the status of interstitial chromosomal loci was ambiguous. Images of wild-type leptotene/zygotene chromosome spreads showing parallel tracks of foci of chromosome axis protein HIM-3 are also highly suggestive of presynaptic alignment [35], but such images lack information regarding chromosome identity, orientation and extent of the proposed alignment. Thus, to directly evaluate the capacity of *C. elegans* chromosomes to achieve full lengthwise alignment in the absence of synapsis, we applied chromosome painting to track chromosome territories in the *syp-1(me17)* mutant, using the three-color chromosome *II* paint introduced in Figure 1.

**Figure 4:**
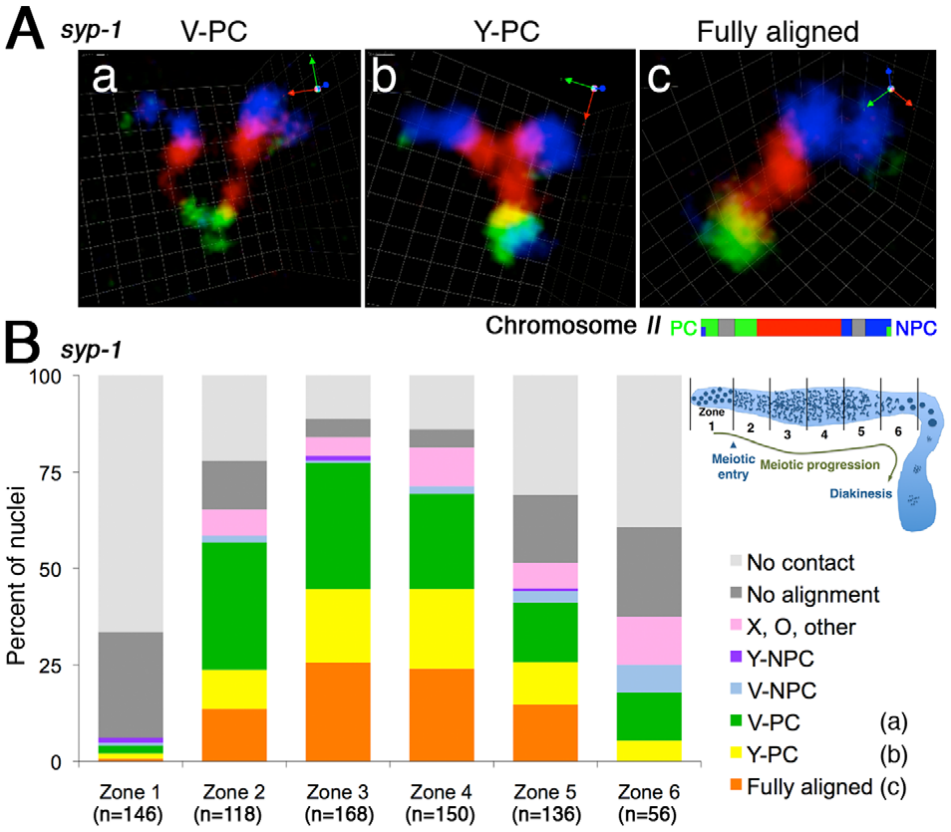
Homologous chromosomes can achieve full alignment in the absence of synaptonemal complex formation. (A) Three-dimensional rendered paint images of the chromosome *II* pair in individual nuclei from the *syp-1(me17)* mutant, which lacks the synaptonemal complex. The probe was identical to that used in Figure 1. Three representative homolog association patterns are shown: (a) V-shaped configuration, with homologs associated only at the left end, which contains the meiotic pairing center (PC); (b) Y-shaped configuration, with homologs aligned and associated from the left (PC) end to the middle of the chromosome; (c) Fully-aligned configuration, in which homologs are aligned and associated along their entire lengths. Scale is shown by the square grid in the background of each panel, with 0.6 µm as the length of each side of the unit square. (B) Quantification of chromosome configurations in *syp-1(me17)* mutant germ lines. As illustrated in the inset diagram, each gonad was subdivided into six zones (one pre-meiotic zone and five equally-sized meiotic zones). For each zone, the fraction of nuclei in which the chromosome *II* pair exhibited the indicated configurations is shown using a stacked bar graph. Nuclei from five gonads were scored for this analysis. In addition to the three categories described in (A), five additional classification categories were used, as follows: V-NPC: V-shaped configuration with homologs associated only at the right (non-PC) end; Y-NPC: Y-shaped configuration with homologs aligned and associated from the right end; X, O, other: X-shaped configuration with homologs aligned and associated only in the middle region, O-shaped configuration with homologs associated only at both ends (but not in the middle region) and other association patterns (*e.g.* double O-shape); No alignment: homologs associated in non-homologous manner, and No contact: No association between homologs.

Figure 4A shows images of the most prominent classes of chromosome association configurations observed during meiotic prophase in the *syp-1* mutant; Figure 4B shows a quantification of the frequencies of these and other association types among nuclei from different zones along the distal/proximal axis of the gonad (representing a time course encompassing meiotic entry and meiotic prophase progression through the end of the pachytene stage). This analysis revealed that when homologous chromosome pairs are associated in the *syp-1* mutant, the two most common configurations are: 1) a “V-shaped” configuration in which homologs are associated only at the end of the chromosomes harboring PC domain (Figure 4A, a), or 2) a configuration in which the homologous territories are closely juxtaposed along their entire lengths (Figure 4A, c). When the two homologous territories are intimately aligned from end to end, they are visualized as a single chromosome territory that is indistinguishable in appearance from the synapsed chromosome pairs observed in wild type nuclei at the pachytene stage (Figure 1h, 1i), with distinct boundaries between chromosomal segments labeled with different fluorophores clearly indicating alignment in register along the entire length of the chromosomes. The high incidence of full alignment demonstrates that *C. elegans* chromosomes have a considerable propensity to align and associate closely along their entire lengths without the aid of SC formation.

Interestingly, there was a relatively low abundance of homolog pairs exhibiting a partial longitudinal association (“Y-shaped” configuration, Figure 4A, b) compared to the V-shaped and fully-aligned configurations. This dearth of intermediate alignment states suggests that the PC-end-only association state and the full alignment state may represent more stable configurations and/or that there may be a relatively rapid transition between them.

### Chromosome territories extend longitudinally after meiotic entry and prior to homolog alignment

**Figure 5:**
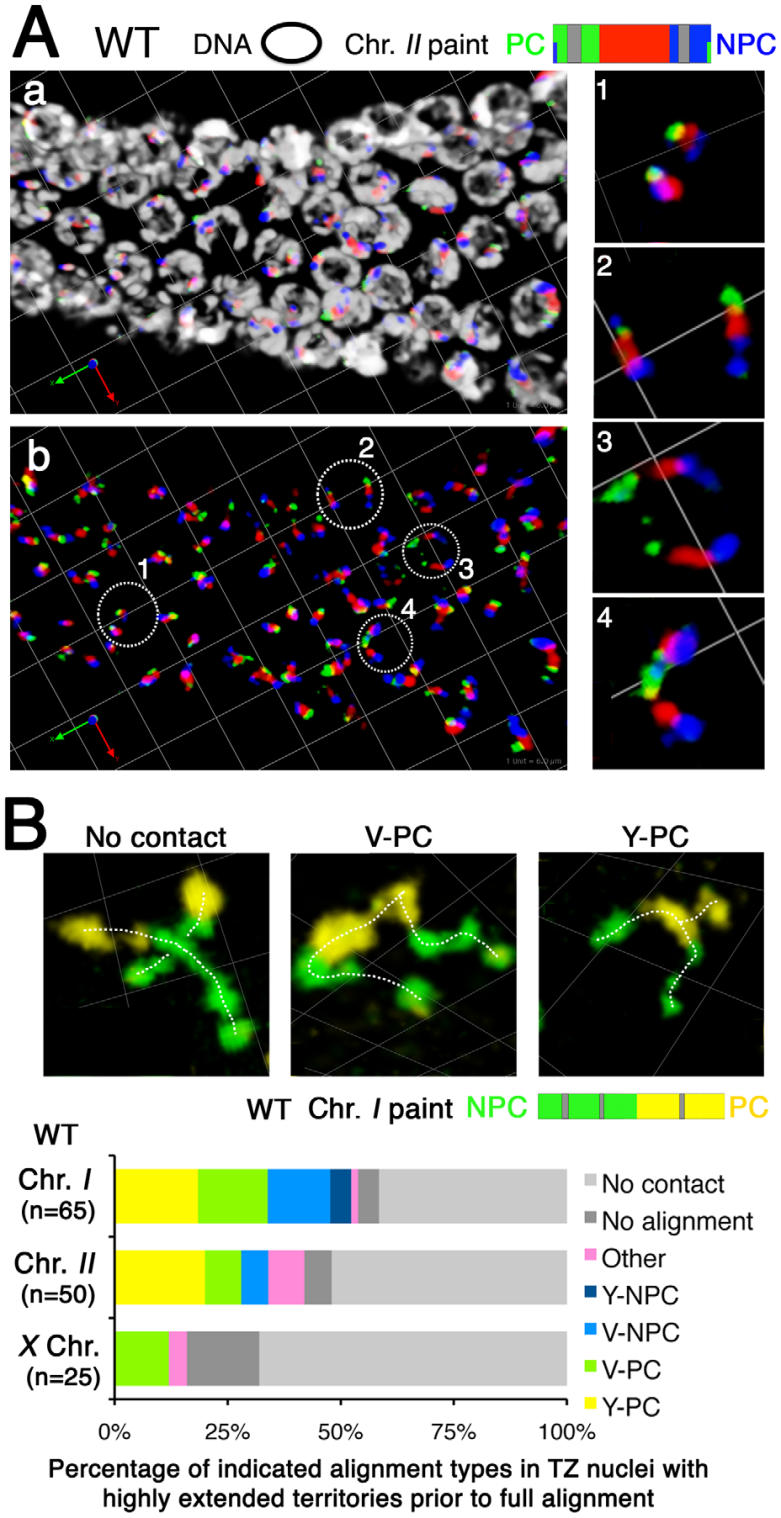
Chromosome extension prior to alignment of homologous chromosomes. (A) Three-dimensional rendered paint images of chromosome *II* in a wild-type germ line. Three fluorophores are assigned as in Figure 1 (Alexa-488, 564 and 647 from the left (PC) end to the right (non-PC) end). DAPI counter stain is white. (a, b) Portion of a gonad spanning the region of meiotic prophase entry. Circles indicate the nuclei shown in close-up at the right. In pre-meiotic nuclei (left side of the image), the chromosome *II* territories are unpaired and compact (close-up 1). Upon entry into the transition zone (in the middle), chromosomes become clustered toward the one side of the nucleus, and longitudinal extension of chromosome *II* territories is observed in a subset of nuclei. Three examples of nuclei with extended territories are shown in close-up: (2, 3) homologous chromosomes with extended territories that are not associated with each other; (4) extended chromosomes partially aligned at the PC end. Scale is shown by the square grid in the background of each panel, with 6 µm as the length of each side of the unit square. (B) Top: Three-dimensional rendered paint images of chromosome *I* in individual nuclei from the transition zone of a wild-type germ line. The left half is painted by Alexa-488 (green) and the right half is painted by Alexa-532 (yellow). White dashed lines are used to trace the paths of the territories. Three different configurations of extended chromosome territories are shown: No contact between homologous territories; Homologous association only at the left end (V-PC); Partial alignment from the left end to the middle of the chromosome (Y-PC). Scale is shown by the square grid in the background of each panel, with 3.8 µm as the length of each side of the unit square. Bottom: Stacked bar graphs showing the distribution of association/alignment states among nuclei with highly extended chromosome territories that do not show full alignment. Nuclei were classified based on the pattern of alignment for chromosome *I*, *II* or *X*. Note that about half show no contact and no alignments between the assayed homologous territories.

Our chromosome painting strategy revealed and allowed us to document an extensive elongation of chromosome territories that occurs in nuclei in the transition zone (Figure 1b–1d; Figure 5). This longitudinal extension is mostly seen after meiotic entry. As shown in Figure 5A, most chromosomes are in compact territories before nuclei enter the transition zone (Figure 5A left, inset 1), but once nuclei are in the transition zone (Figure 5A, a, b middle), longitudinal extension of chromosome territories is clearly observed in a subset of nuclei (Figure 5A, insets 2–4). For purposes of quantitation, we defined “highly extended” chromosome territories as those exhibiting an extremely elongated thread-like morphology that was clearly distinguishable from the compact ovoid shape of territories in the pre-meiotic region. Such territories have a slenderness ratio (the ratio of the approximate length to the approximate width of the painted chromosome) of >6. Half of chromosome *I* territories and 25–30% of *X* chromosome territories were scored as “highly extended” in wild-type transition zone nuclei (see below).

Our analysis indicates that longitudinal extension of chromosome territories initiates before homologous association. Among nuclei exhibiting highly extended chromosome territories prior to full alignment, there was no obvious relationship between the elongation state and the association status of a homolog pair; extended chromosome territories were seen for non-associated, end-associated and partially aligned chromosomes (Figure 5B). Quantitation of association/alignment states in these nuclei revealed that between 42% (chromosome *I*) and 68% (*X* chromosomes) of such nuclei showed no evidence of homologous association between the assayed chromosome pair. Therefore, neither alignment nor association between homologous chromosomes is a prerequisite for the longitudinal extension of chromosome territories. This observation indicates that chromosome territories extend from a compact shape to an elongated shape in early meiotic prophase, before initiating homologous alignment.

Among the chromosome *I* and chromosome *II* territories analyzed in Figure 5B, association of chromosome territories only at the PC end or only at the non-PC end (V-PC or V-NPC configuration) was observed with similar frequencies. In contrast, partial alignment of chromosome territories along the PC half of the chromosome (the Y-PC configuration) occurred much more frequently than the Y- NPC configuration (which was rarely observed). This observation suggests that even though the non-PC terminus of a chromosome has some potential to engage in homologous association very early in meiotic prophase, this activity is much weaker than the robust pairing-stabilization activity at the PC terminus, which is capable of propagating pairing into adjacent chromosome regions.

**Figure 6:**
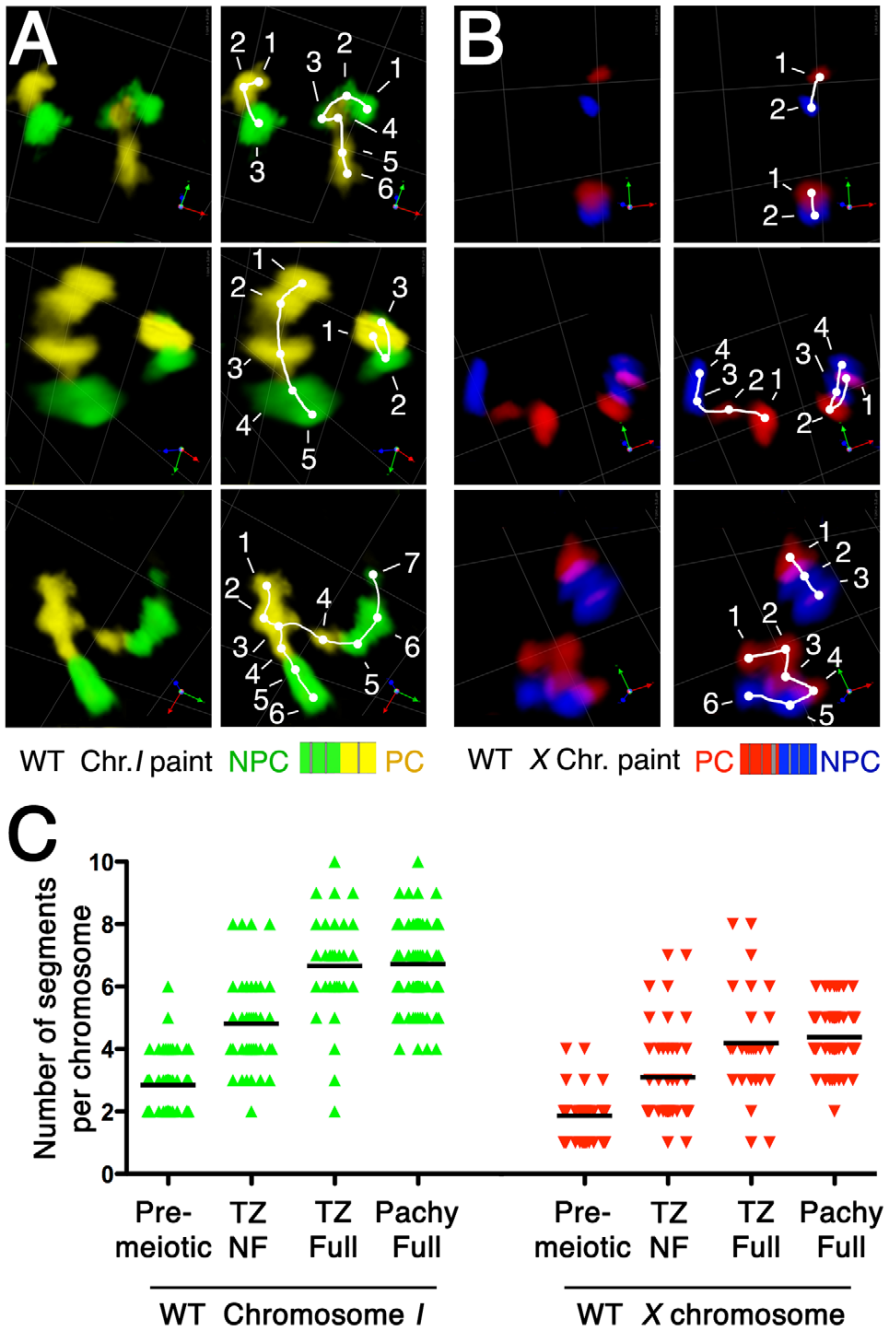
Increase in number of painted chromosome segments accompanies elongation of chromosome territories. (A and B) Pairs of images depicting quantitation of painted chromosome segments, which increase in number in parallel with longitudinal extension of chromosome territories. (A) The left half of chromosome *I* is painted by Alexa-488 (green) and the right half is painted by Alexa-532 (yellow). (B) The left half of the *X* chromosome is painted by Alexa-594 (red) and the right half is painted by Alexa-647 (blue). For each nucleus depicted in A and B, in the right-hand panels, visually discernable painted chromosomal segments are marked with dots, which are numbered sequentially and are connected by a line tracing the path of the chromosome territory. Scale is shown by the square grid in the background of each panel, with 3.8 µm as the length of each side of the unit square. (C) Quantification of the numbers of painted chromosome segments in nuclei from the pre-meiotic zone, the transition zone (TZ) and the early pachytene zone (Pachy Full). The chromosome territories in the transition zone were subdivided into two groups based on the status of alignment: fully aligned (TZ Full) and not-fully aligned (TZ NF). The numbers of painted segments per chromosome in individual nuclei of each sample are displayed as scatter plots, with a horizontal black line showing the average.

Elongation of chromosome territories revealed a beads-on-string appearance of the chromosome paint signals: chromosomal segments of high signal intensity are interspersed with regions of low intensity or gaps. Further, while the number of “beads” depends on the composition of the probe, an increase in the degree of extension generally correlates with an increase of the number of discernable painted segments per chromosome (Figure 6A, 6B). Thus, counting of painted segments provides a means to quantify and compare the elongation states of the chromosomes at different stages. Specifically, we counted the numbers of these “painted segments” in three-dimensionally rendered images, as illustrated in Figure 6A, 6B (see Videos S1 and S2 and Materials and Methods). This analysis was conducted for chromosome *I* and for the *X* chromosome, for nuclei from three distinct zones within the gonad: the pre-meiotic zone, the transition zone, and the early pachytene zone. Further, for plotting of the data (Figure 6C) we sorted nuclei from the transition zone into two classes: those that had not yet achieved full alignment (TZ-NF), and those that had achieved full alignment (TZ-Full, which likely includes nuclei that had reached the pachytene stage). This quantitative treatment provided strong statistical support for the observations described above. For both chromosome *I* and *X*, there was an extremely significant increase in the number of painted chromosomal segments between pre-meiotic nuclei and TZ-NF nuclei (p<0.0001), reflecting a substantial elongation of chromosome territories following meiotic entry. Moreover, we also detected an extremely significant increase in the number of painted segments per chromosome between TZ-NF nuclei and TZ-Full nuclei (p<0.0001). This increase indicates an ongoing elongation of chromosome territories within the TZ as homolog alignment proceeds to completion.

In contrast, we did not detect a significant difference in the number of painted chromosomal segments between TZ-Full and pachytene nuclei for either chromosome. In part, this observation likely reflects inclusion of some pachytene nuclei in the TZ-Full category. However, it also suggests that while the segment-counting assay clearly reports on one aspect of chromosome elongation, it may not capture the full extent of pachytene chromosome extension.

### Longitudinal extension of chromosome territories occurs in mutants defective for SC assembly and chromosome dynamics mediated by PC–SUN-1/ZYG-12 linkages

**Figure 7:**
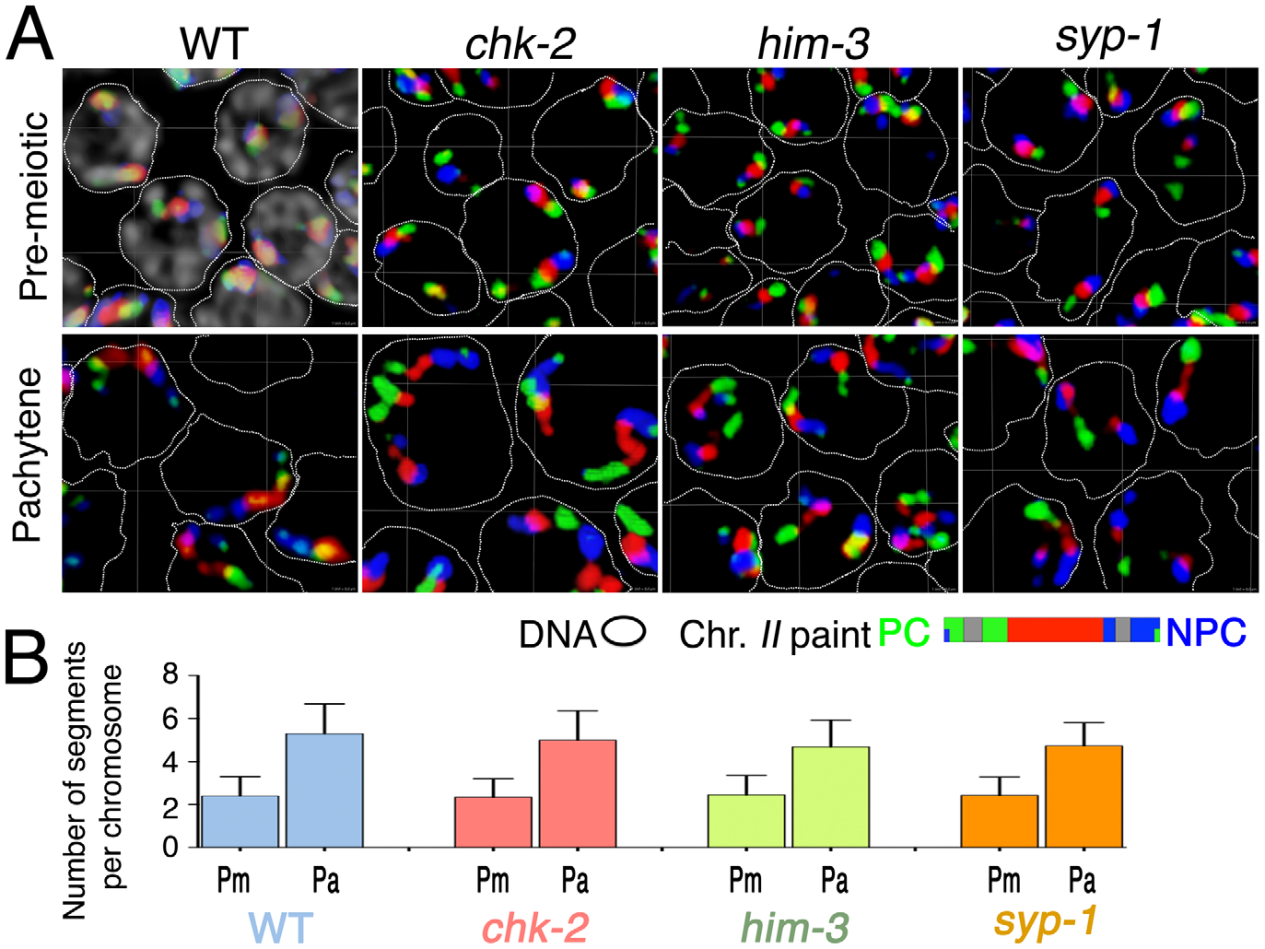
Longitudinal extension of chromosome territories in mutants defective for SC assembly and chromosome dynamics mediated by PC–SUN-1 linkages. (A) Three-dimensional rendered paint images of chromosome *II* in premeiotic and early pachytene nuclei from wild-type and *chk-2(me64)*, *him-3(gk149)* and *syp-1(me17)* mutant germ lines. Three fluorophores are assigned as in Figure 1 (Alexa-488, 564 and 647 from the left (PC) end to the right (non-PC) end). In the panel showing wild-type pre-meiotic nuclei, DAPI counter stain is shown in white and a dotted line is used to encircle the domain of DAPI-stained chromatin within each nucleus; in other panels, DAPI is omitted to facilitate visualization of the paint signals. Note that chromosome *II* territories show obvious longitudinal extension in all of these strains. Scale is shown by the square grid in the background of each panel, with 6 µm as the length of each side of the unit square. (B) Numbers of painted segments per chromosome scored for chromosome *II* in nuclei from the pre-meiotic zone (Pm) and the early pachytene zone (Pa) of germ lines of the indicated genotype. For each genotype, nuclei from two gonads were scored; error bars indicate standard deviation.

The timing of the longitudinal extension coincides temporally with a period of active chromosome movement that is mediated by association of chromosome pairing centers (PCs) with mobile patches of the NE-spanning SUN-1/ZYG-12 protein complexes. In order to test whether PC–SUN-1/ZYG-12-mediated movement of chromosomes is required for chromosome elongation, we evaluated territory elongation in mutants defective for *chk-2* (which encodes a protein kinase required for homolog pairing and nuclear reorganization during meiotic prophase [30]), *him-3* (which encodes a major chromosome axis component required for synapsis [36]), and *syp-1* (which encodes a major component of the SC central region [19]. Previous work has shown that: 1) NE-associated PC–SUN-1/ZYG-12 aggregates do not form and SUN-1 is not phosphorylated in *chk-2* mutants [22], [26]; 2) *him-3* mutants are defective for formation of autosomal PC–SUN-1/ZYG-12 aggregates [37]; and 3) in a *syp* mutant, the movement of SUN-1 aggregates is slower and more spatially constrained than during wild-type meiosis [37]. We used three-color paints to assess chromosome *II* territory morphology in pre-meiotic nuclei and in nuclei within an early meiotic prophase zone (between 40 and 50 rows from the distal tip) that corresponds to early pachytene in the wild type controls. The images shown in Figure 7A and the quantitation of painted chromosome segments in Figure 7B both indicate that substantial elongation of chromosome *II* territories occurs in all of these mutants. *chk-2* mutants exhibited an extremely significant increase in the number of painted chromosomal segments (p<0.0001) between the premeiotic and pachytene stages. Moreover, the number of painted segments in pachytene nuclei in the *chk-2* mutant was indistinguishable from wild-type controls (p = 0.122), suggesting a normal degree of territory elongation. *him-3* and *syp-1* mutants similarly exhibited extremely significant increases in the number of painted segments between the premeiotic and pachytene stages (p<0.0001); however, these mutants had modest but significant differences from wild type at the pachytene stage (p = 0.0006), suggesting a slight reduction in the extent of elongation.

The apparently normal chromosome elongation observed in the *chk-2* mutant indicates that phosphorylation of SUN-1 and association of PCs with mobile SUN-1/ZYG-12 patches at the NE are not required to accomplish elongation of chromosome territories during early meiotic prophase. This in turn implies that chromosome movements mediated by PC–SUN-1 linkages are not the primary driver of territory elongation. Further, the substantial territory elongation observed in the *him-3* and *syp-1* mutants also indicates that a high degree of elongation can be achieved without SC assembly.

### PC binding protein HIM-8 is required for normal elongation of *X* chromosome territories

**Figure 8:**
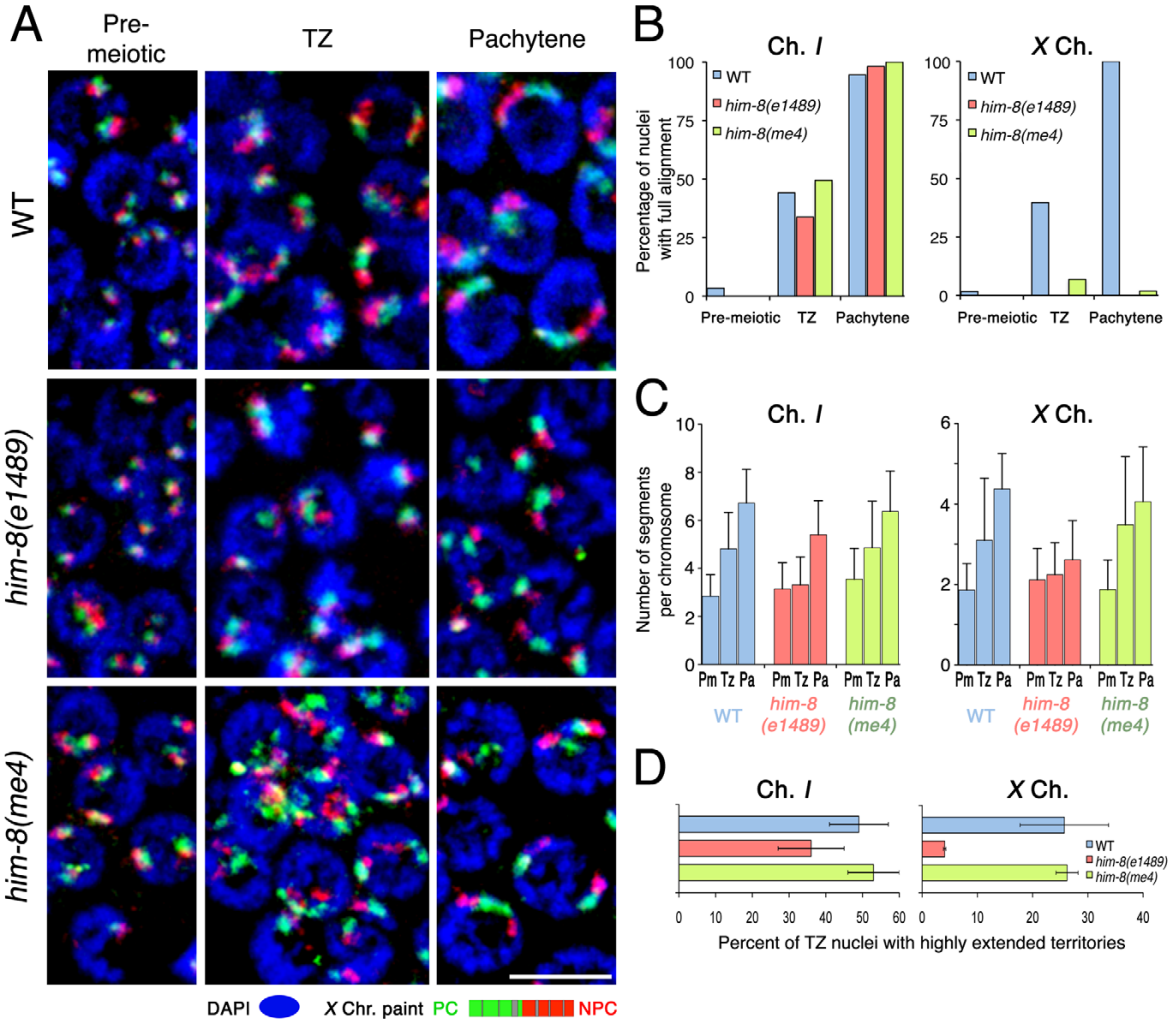
Roles for pairing center binding protein HIM-8 in elongation of *X* chromosome territories. (A) Maximum intensity projections of paint images of the *X* chromosomes in nuclei from the indicated regions from wild type (upper), *him-8(e1489)* (middle) and *him-8(me4)* (bottom) germ lines. The *X* chromosome is painted with Alexa-594 (green) in the left half and Alexa-647 (red) in the right half. DAPI is in blue. Note that the *him-8*(*e1489*) mutant fails to show longitudinal extension either in the transition zone or at the pachytene stage, whereas *him-8*(*me4*) shows longitudinal extension of *X* at both stages (as in wild type) despite the fact that *X* chromosomes fail to align. (B) Percentage of nuclei that show full alignment of chromosome *I* (left) and *X* (right) in the pre-meiotic zone, the transition zone and the pachytene zone. Chromosome *I* fully aligns in both *him-8*(*e1489*) and *him-*8(*me4*) mutant as in the wild type, whereas the *X* chromosomes do not show any significant full alignment in either *him-8* mutant. Scale Bar: 5 µm. (C) Numbers of painted segments per chromosome scored for chromosome *I* (left) and *X* (right) in nuclei from the pre-meiotic zone (Pm), the transition zone (Tz) and the pachytene zone (Pa) of germ lines of the indicated genotype. For the transition zone, only those nuclei that had not achieved full alignment were included in this analysis (see Figure 3D). For each genotype, nuclei from three gonads were scored; error bars indicate standard deviation. (D) Percentage of transition zone nuclei exhibiting highly extended chromosome territories. Chromosome territories were scored as “highly extended” if they exhibited an elongated thread-like morphology that was clearly distinguishable from the compact ovoid shape characteristic of pre-meiotic nuclei; these chromosomes had a slenderness ratio of >6. For each of the indicated genotypes, nuclei from three gonads were scored; error bars indicate standard error of the mean.

Although chromosome movement mediated by PC–SUN-1/ZYG-12 linkages does not seem to be required for the territory elongation, PCs have been shown previously to play several distinct roles in promoting homolog synapsis. Therefore, we tested the possibility that PCs might contribute to elongation in other ways. Specifically, we investigated the importance of *X* chromosome PC function in the process of chromosome territory elongation. We first evaluated the potential contribution of HIM-8, a zinc-finger protein that concentrates at the PC domain of the *X* chromosome and is required for PC function [21], by assessing *X* chromosome territory elongation in *him-8* mutants. Because impaired *X* pairing in *him-8* mutants results in an expanded zone of nuclei with a clustered chromosome distribution, we used the alignment status of chromosome *I* to define the boundary between transition zone and pachytene for this analysis. Specifically, we defined the transition zone as the region of the gonad in which nuclei exhibited a clustered distribution of chromosomes and a mixture of unaligned, partially aligned and fully aligned chromosome *I* territories (Figure 8B). The length of the transition zone defined by these criteria is very similar among the strains tested (Materials and Methods and Figure S1). Nuclei in the pachytene zone, adjacent to the transition zone, mostly contained fully aligned chromosome *I* territories.

In wild type controls, *X* chromosome territories extended longitudinally in the transition zone (Figure 8A), which was quantitatively reflected as an increase of the number of painted chromosomal segments (Figure 8C; p<0.0001). In the pachytene zone, we saw full alignment of *X* chromosomes (Figure 8A, 8B) and a further elongation of the *X* chromosome territory (Figure 8A), reflected by a corresponding increase in the number of painted segments (Figure 8C; p<0.0001). In contrast, we did not observe any obvious longitudinal extension of *X* chromosome territories in transition zone nuclei in the *him-8* (*e1489*) mutant (Figure 8A), which is a putative null allele that encodes a mutant HIM-8 protein with a disrupted DNA binding domain that does not localize to the *X* chromosomes [21]. A severe impairment of *X* chromosome elongation was clearly reflected both in quantitation of painted chromosomal segments, which did not detect any significant difference between premeiotic and transition zone nuclei in the *him-8*(*e1489*) mutant (Figure 8C; p = 0.27), and in the frequency of transition zone nuclei exhibiting highly extended *X* chromosome territories (Figure 8D), which was greatly reduced relative to the wild-type control (p<0.0001). Chromosome territories also remained relatively compact in the pachytene region of *him-8(e1489)* mutant germ lines, even at the late pachytene stage where extension of chromosome territories is most obvious in wild-type germ cells (Figure 8A and data not shown). Further, although we did detect a modest increase in the number of painted segments per chromosome between transition zone and pachytene nuclei in the *him-8(e1489)* mutant (Figure 8C; p = 0.014), the magnitude of this increase was quite small in comparison to controls. The impairment of *X* chromosome territory elongation in the *him-8*(*e1489*) mutant clearly indicates that HIM-8 is required for this elongation.

We also examined *X* chromosome elongation in the *him-8(me4)* mutant. *him-8(me4)* produces a mutant HIM-8 protein that retains its ability to localize onto the *X* chromosomes and to the NE, but does not support HIM-8 functions in promoting pairing and SC formation and is deficient for coupling of the *X*-PCs to mobile SUN-1/ZYG-12 patches [21], [25]. In striking contrast to the *him-8*(*e1489*) mutant, *X* chromosome territory elongation was normal in the *him-8*(*me4*) mutant. Successful *X* territory elongation in the *him-8*(*me4*) mutant was clearly evident in the images in Figure 8A and is reflected both in an increased number of painted chromosomal segments (Figure 8C, showing that the data for *him-8*(*me4*) closely parallel the control data) and in the frequency of transition zone nuclei exhibiting highly extended *X* chromosome territories (Figure 8D). Whereas *X* chromosome elongation was normal in this mutant, we confirmed the previously reported failure in *X* chromosome alignment (Figure 8B). Thus, taken in the context of prior work indicating failure in coupling to SUN-1/ZYG-12 in the *him-8(me4)* mutant [25], our results indicate that the function of HIM-8 in promoting *X* chromosome elongation is distinct and genetically separable from its role in linking the *X* chromosome PC to the cytoskeletal motility apparatus through the known mechanism mediated by SUN-1/ZYG-12 patches. As motility of the *X*-PC has not been assessed directly in the *him-8*(*me4*) mutant, we cannot exclude the possibility of *X* chromosome movement by an alternative mechanism. However in the context of our analysis of *chk-2*, *him-3* and *syp-1* mutants, our analysis of the *him-8*(*me4*) mutant provides additional support for the conclusion that chromosome elongation is not merely a secondary consequence of PC–SUN-1/ZYG-12-mediated chromosome mobilization.

### Deletion of the *X*-PC leads to partial impairment in elongation of *X* chromosome territories

**Figure 9:**
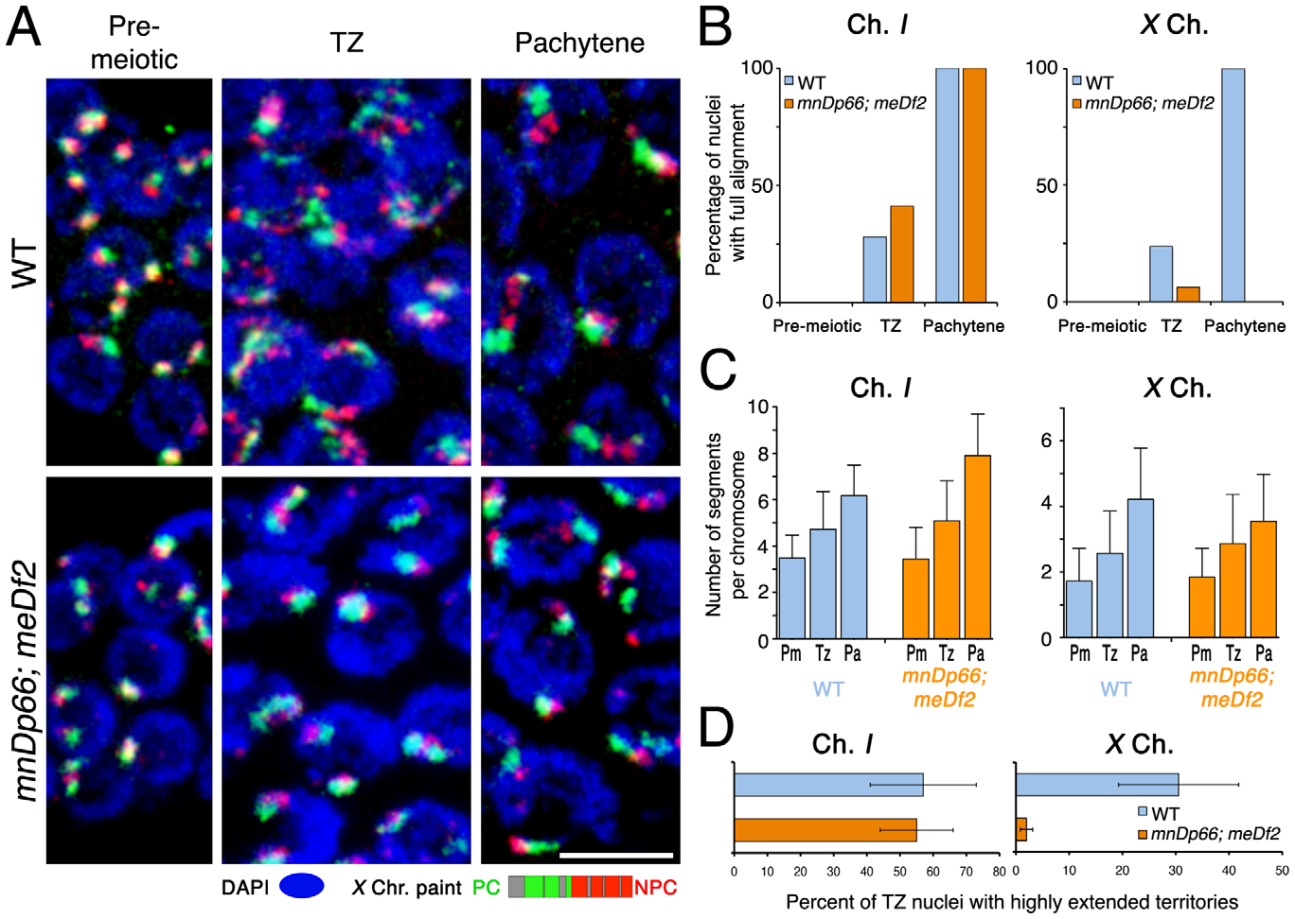
Pairing centers are required for normal longitudinal extension of the *X* chromosome territories. (A) Maximum intensity projections of paint images of normal *X* chromosomes or *X* chromosomes with the *meDf2* deletion (which removes the *X*-PC), in nuclei from the indicated germline zones. As indicated in the schematic, the paint probe is similar to the *X* paint used in other analyses, except that it does not include YACs corresponding to the region deleted by *meDf2*. Note that the *meDf2 X* chromosomes do not show obvious longitudinal extension either in the transition zone or at the pachytene stage. However, in contrast to the *him-8(e1489)* mutant (Figure 8A), *X* territories in *meDf2* homozygotes do have a beaded appearance, and an increase in the number of painted segments per chromosome is detected. Scale Bar: 5 µm. (B) Percentage of nuclei that show full alignment of chromosome *I* (left) and *X* (right) in the pre-meiotic zone, the transition zone and the pachytene zone. In *meDf2* germ lines, chromosome *I* fully aligns as in the wild type but the *X* chromosomes do not show any significant full alignment. (C) Numbers of painted segments per chromosome scored for chromosome *I* (left) and *X* (right) in nuclei from the pre-meiotic zone (Pm), the transition zone (Tz) and the pachytene zone (Pa) of germ lines of the indicated genotype. For the transition zone, only those nuclei that had not achieved full alignment were included in this analysis (see Figure 3D). For each genotype, nuclei from three gonads were scored; error bars indicate standard deviation. (D) Percentage of transition zone nuclei exhibiting highly extended chromosome territories. Chromosome territories were scored as “highly extended” if they exhibited an elongated thread-like morphology that was clearly distinguishable from the compact ovoid shape characteristic of pre-meiotic nuclei (slenderness ratio >6). For each of the indicated genotypes, nuclei from three gonads were scored; error bars indicate standard error of the mean.

We further investigated the role of the *X* chromosome PC in chromosome territory extension using a strain homozygous for an *X* chromosome that lacks the PC region, *meDf2* [18]. To conduct an appropriate direct comparison between the behavior of full-length *X* chromosomes and *meDf2 X* chromosomes, we generated a paint probe that did not include the portions of the *X* that are deleted by *meDf2*. As for the *him-8* experiments, we defined the transition zone and the pachytene zone using DAPI staining and chromosome *I* alignment as reference points (see Figure S1 and Materials and Methods). In the wild type, *X* chromosome territory elongation was successfully detected with this paint probe, which was obvious in the appearance of chromosome territories in the transition zone and pachytene zone (Figure 9A top panels), in corresponding increases in the number of painted segments per chromosome (Figure 9C), and in a frequency of transition zone nuclei exhibiting highly extended *X* chromosome territories that was comparable to that detected using a paint probe representing the complete *X* chromosome (Figure 8D, Figure 9D).

**Figure 10:**
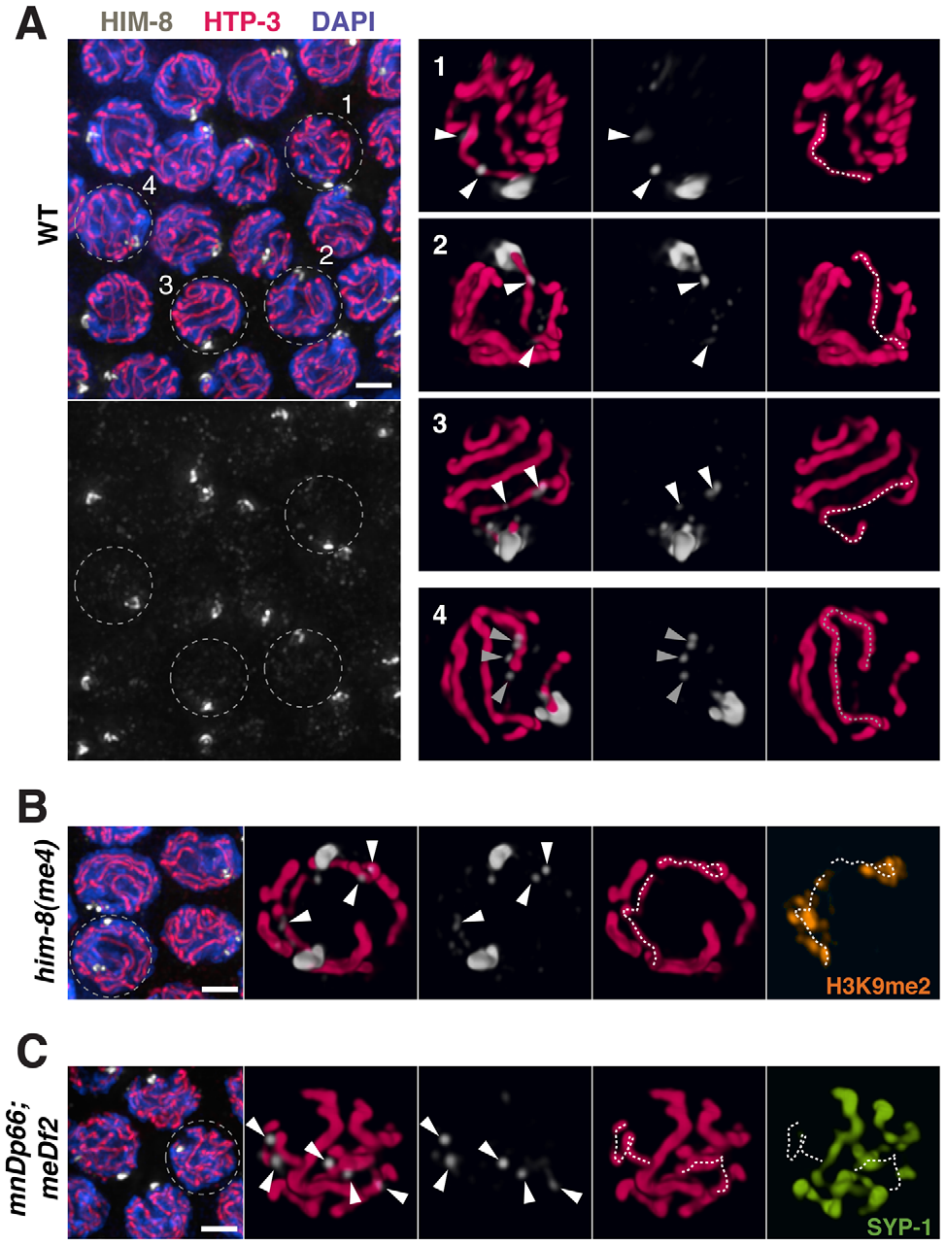
HIM-8 localizes to chromosome sites outside the *X* Pairing Center. (A) Mid-pachytene nuclei in wild type show one major focus of HIM-8 (white) staining corresponding to the paired *X* PCs, as well additional fainter HIM-8 speckles elsewhere in the nucleus. Images on the left are projections of deconvolved optical sections spanning the full nuclear depth. Scale bar, 2 µm. Images on the right are 3-D surface renderings comprising half the depths of nuclei 1–4. These images show HIM-8 speckles localizing to discrete sites along the lengths of the *X* chromosome and autosomes, with paths of the chromosomes visualized by immunostaining of chromosome axis marker HTP-3 (pink). While there is generally at least one speckle adjacent to each chromosome axis, the most prominent HIM-8 speckles are found along the axis of the *X* chromosome and at one end of an autosome. Nuclei 1–3 show the typical localization of two prominent HIM-8 speckles (white arrowheads) along the axis of the *X* chromosome (white dotted trace); 3 or more prominent speckles are sometimes detected on the *X*. Nucleus 4 shows a cluster of HIM-8 speckles (grey triangles) characteristic of one end of an autosomal axis (grey dotted trace). (B) Analysis of HIM-8 localization in the *him-8(me4)* mutant. Mid-pachytene nuclei show two major foci of HIM-8 marking the unpaired *X* PCs; in this field, the HIM-8 foci are well separated in the Z dimension in all nuclei. Scale bar, 2 µm. The indicated nucleus is depicted in 3-D as in (A). HIM-8 speckles localize adjacent to the axes of the unpaired *X* chromosomes, which are highlighted by immunostaining of H3K9me2 (orange), which concentrates on unsynapsed *X* chromosomes [48]. (C) Analysis of HIM-8 localization in the *meDf2* homozygote. Mid-pachytene nuclei show one major focus of HIM-8 staining corresponding to the paired *mnDp66* chromosomes, which contain a copy of the *X*-PC region fused to chromosome *I*. The *meDf2 X* chromosomes, which lack the *X*-PC and thus do not have an associated major HIM-8 focus, are unpaired. Scale bar, 2 µm. The indicated nucleus is depicted in 3-D as in (A). (Note: the major HIM-8 focus on the *mnDp66* chromosome is in the half of the nucleus not depicted in the 3-D rendering.) The axes of the unpaired *X* chromosomes are identified by lack of associated immunostaining for SC central region protein SYP-1 (green). Although these *X* chromosomes lack PCs and the associated major HIM-8 foci, HIM-8 speckles still localize along the unpaired *X* chromosome axes. In addition, the paths of the *X* chromosome axes in this nucleus are coiled (left) or folded (right), although they appear comparable in length to those seen in the *him-8(me4)* mutant. This type of organization is common for *X* chromosomes in *meDf2* homozygotes and may account for the fact that *meDf2 X* chromosomes have relatively compact ovoid territories despite exhibiting increases in the number of discernable painted chromosome segments.

The *meDf2* homozygote exhibited a distinct phenotype in which the *X* chromosomes were intermediate in character between the wild-type full length *X* chromosomes and those in the *him-8*(*e1489*) mutant. On the one hand, *meDf2* homozygotes exhibited increases in the numbers of painted *X* chromosome segments that paralleled those observed in wild-type controls (Figure 9C), reflecting significant remodeling of X territories. However, *X* chromosome territories had an overall more compact appearance in *meDf2* homozygotes than in controls (Figure 9A), and the frequency of transition zone nuclei with highly extended *X* chromosome territories was greatly reduced (Figure 9D; p<0.0001), to a degree comparable to that seen in the *him-8(e1489)* mutant (Figure 8D). These features may reflect the *meDf2* chromosomes having a more folded or coiled organization, as suggested by the images in Figure 10C and Figure S2. We interpret these data as reflecting a partial impairment of *X* chromosome territory elongation in *meDf2* homozygotes.

The observed partial impairment of *X* chromosome territory elongation in *meDf2* homozygotes is quite distinct from the phenotype of *him-8*(*e1489*) mutants, in which all aspects of *X* territory restructuring are profoundly impaired. This discrepancy in phenotype suggests that HIM-8 protein may influence the behavior of *meDf2 X* chromosomes even though they lack a PC. That is, in addition to functioning in association with the PC, HIM-8 protein may interact with the *X* chromosomes in regions outside the PC to influence reorganization of chromosome structure during meiotic prophase. Several prior studies have suggested function of HIM-8 outside of the *X*-PC: *meDf2* homozygotes show higher frequencies of bivalent formation and homolog synapsis and lower *X* chromosome missegregation compared to *him-8* loss-of-function mutants [18], [21], [38], HIM-8 consensus binding sites are present along the *X* chromosome as well as at lower levels on the autosomes [23], and genetic interactions between *him-8* and several transcription factors have raised the possibility that HIM-8 might play a broader role in influencing chromatin state [39]. Actual localization of HIM-8 outside of the *X*-PC, however, has not been demonstrated. Therefore, we carefully re-examined HIM-8 localization by immunofluorescence. In addition to the major focus of HIM-8 at the *X*-PC, we detected lower levels of HIM-8 at a number of additional chromosome sites (Figure 10A). Additional HIM-8 signals were most prominent on the *X* chromosomes, but were also detected on autosomes. HIM-8 was also observed at non-PC regions of the unpaired *X* chromosomes in *him-8(me4)* mutants and in *meDf2* homozygotes (Figure 10B, 10C). These localization data are consistent with the possibility that HIM-8 may promote remodeling of meiotic chromosome structure through association with multiple sites along the lengths of chromosomes.

## Discussion

### Visualization of whole chromosome territories constrains models for mechanisms that promote chromosome sorting

Elucidation of the early events that enable homologs to locate and recognize their appropriate pairing partners has been hindered in part by limited knowledge about how chromosomes are organized within nuclei at the relevant stages. By enabling visualization of whole chromosome territories both prior to meiosis and during early meiotic prophase, our chromosome painting analysis has allowed us to constrain our thinking regarding potential mechanisms that might contribute to chromosome sorting.

While it was clear from prior analysis that homologs achieve *de novo* alignment during *C. elegans* meiosis [29], the possibility remained that homologous territories might exhibit preferential proximity (albeit without alignment) that could facilitate chromosome sorting. Without knowledge about the shape and spatial arrangement of premeiotic chromosome territories, this hypothesis could not be excluded based on existing data. Our finding that homologous chromosome territories are frequently quite distant from each other prior to meiotic entry rules out models in which premeiotic proximity is the primary driver of homolog sorting. This is an important finding in light of the fact that *C. elegans* has a robust capacity to achieve homologous synapsis in the absence of meiotic recombination [29]. This property is shared with *Drosophila* females [40] but appears to be a derived trait, as the phylogenetic distribution of recombination-dependence for SC formation (and of proteins relevant for this process) [41] suggest that a recombination-based mechanism for homology verification is the ancestral state. Whereas premeiotic colocalization of homologs [42], [43] appears to be at least part of the explanation for loss of reliance on recombination in *Drosophila*, it is clear that *C. elegans* requires mechanisms that can bring distantly located homologs into proximity.

Although our data exclude premeiotic proximity as a primary driver of chromosome sorting, it is possible that the spatial organization of premeiotic nuclei may contribute to pairing of the *X* chromosomes. While *X* chromosomes were no more likely than autosomes to be closely associated with their homologs, they were significantly less likely to be closely associated with a heterologous chromosome, in a manner dependent on HIM-8. We suggest that a propensity to avoid inappropriate contacts could indirectly facilitate interactions with an appropriate partner.

Our data also argue against the possibility that temporal heterogeneity in the behavior of chromosomes might serve as a primary driver of chromosome sorting. Under this type of scenario, different chromosomes would differ in the timing with which they acquire competence for interchromosomal interactions. A key prediction of this model is that chromosomes would exhibit a clear temporal hierarchy in achieving homolog alignment. As no such hierarchy was observed, this type of mechanism does not appear to be a major factor contributing to homolog recognition in *C. elegans*.

Whereas we did not find evidence for either premeiotic proximity or temporal heterogeneity in chromosome behavior as key mechanisms underlying homolog pairing, our painting analysis did reveal several features that could contribute: 1) a capacity for synapsis-independent full-length alignment of homologs, 2) synapsis-independent restructuring and elongation of chromosome territories, and 3) for the *X* chromosomes, dependence of territory remodeling on the *X*-PC and HIM-8. We consider the implications of these findings below.

### Roles for HIM-8 and the *X*-PC in meiotic prophase remodeling of chromosome territories

In our analysis of wild-type *C. elegans* germ lines, we found that chromosome territories are dramatically remodeled at the onset of meiotic prophase. The relatively compact ovoid territory organization present in premeiotic germ cells is transformed into a longitudinally extended, threadlike organization. This transformation is apparent prior to association and alignment of homologous chromosomes. Similar elongation of chromosome territories has been observed in meiocytes of yeast [44], [45], tetrahymena [46], maize [47], oat/maize hybrids [9], wheat/rye hybrids [11]–[13] and human [6], suggesting that territory elongation is a conserved feature of the meiotic program. Our ability to visualize this conserved feature in *C. elegans* provides an excellent opportunity to investigate its mechanistic basis.

Our analysis revealed that normal remodeling of *X* chromosome territories depends on the function of both the *X*-PCs and *X*-PC binding protein HIM-8. Moreover, our images reveal a difference between the contributions of the PC *per se* and the HIM-8 protein to altering chromosome architecture, implying that HIM-8 can function outside of the PC to mediate a component of this restructuring. In wild-type germ cells, longitudinal extension of chromosome territories is associated with an increase in the number of discernable painted segments per chromosome. *X* chromosome territory elongation is profoundly impaired in the *him-8*(*e1489*) mutant (in which no HIM-8 protein is detected on chromosomes [21]), indicating that HIM-8 plays a central role in chromosome restructuring. However, analysis of chromosomes deleted for the *X*-PC showed that chromosome territories can remain relatively compact and ovoid despite an increase in the number of painted segments per chromosome. This suggests that these chromosomes are competent to undergo partial elongation, but cannot achieve full longitudinal extension of their territories, likely because the chromosomes remain in a partially coiled or folded state. These data reveal a difference in the contributions of HIM-8 and the *X*-PC to *X* chromosome territory elongation, helping to reconcile previously unexplained but reproducible observations that *him-8* null mutants show higher frequencies of *X* chromosome missegregation than *X*-PC deletion homozygotes [18], [21]. We suggest that while HIM-8 protein concentrated at the PCs is essential to achieve maximal longitudinal extension of *X* territories, HIM-8 binding to non-PC sites elsewhere on the *X* chromosome can promote a degree of territory elongation that manifests as an increase in the number of painted segments.

In addition to providing evidence that HIM-8 functions both at and outside the PC to promote *X* chromosome restructuring, our analysis also demonstrated that the role(s) for HIM-8 and the *X*-PC in restructuring of *X* chromosome territories are distinct and separable from HIM-8/PC function in SUN-1/ZYG-12-mediated chromosome mobilization. We found that the mutant HIM-8^me4^ protein, which binds to *X* chromosomes [21], is able to promote territory elongation despite its inability to support association of the *X*-PCs with SUN-1/ZYG-12 patches. Furthermore, we demonstrated that chromosome *II* territory elongation appears normal in the *chk-2* mutant, which is defective for both the phosphorylation of SUN-1 and the formation of PC–SUN-1/ZYG-12 aggregates that are associated with chromosome movement and homolog pairing. Together these data argue that PC–SUN-1/ZYG-12-mediated chromosome movement is not the primary driving force responsible for elongation of chromosome territories.

It remains an open question whether autosomal PCs and/or PC-binding proteins, ZIM-1,-2, -3 [22], similarly function in promoting restructuring of chromosome territories. Our initial analysis of *zim-1* and *zim-3* mutants did not uncover an obvious impairment of territory elongation (K.N., unpublished), which may indicate that different mechanisms underlie elongation of the *X* chromosomes and autosomes. It is possible that the *X* chromosomes, which are largely transcriptionally quiescent in the germ line [48], might require a special mechanism to promote elongation in order to counteract an inherent tendency to adopt a compact territory organization. Alternatively, the ZIM proteins may contribute to restructuring of autosomes, but there may be substantial redundancy among the HIM-8/ZIM protein family in fulfilling this role.

### Integrating a gallery of painting data into a model for homolog pairing

Our imaging of chromosome territories in *syp-1* mutants, where early pairing intermediates cannot be stabilized by synapsis, provides additional insight regarding how productive homolog alignment may be achieved during *C. elegans* meiosis. Chromosome painting revealed three predominant modes of homolog association in the *syp-1* mutant: V-PC (associated only at the PC end), Y-PC (close association along part of the chromosome, including the PC end), and full lengthwise alignment. The high incidence of the V-PC and Y-PC configurations reinforces the previous conclusion that PCs have a robust capacity to confer local stabilization of pairing in the absence of synapsis. Moreover, the prevalence and relative abundance of these three configurations allows several additional inferences regarding the nature of synapsis-independent interhomolog interactions.

First, the substantial fraction of homolog pairs in the “full alignment” category clearly establishes that *C. elegans* chromosomes can achieve full lengthwise alignment independently of synapsis. Whereas full-length alignment of homologs independent of SC assembly had been demonstrated previously for a variety of organisms in which SC formation is coupled to initiation of interhomolog recombination (*e.g.* [49]–[51], reviewed in [52], [53]), our data show that SC-independent full-length alignment also occurs in an organism where homologous synapsis does not depend on recombination. Multicolor paint shows different colored segments in register in the “full alignment” configuration, indicating that this organization does not simply represent coincidental colocalization resulting from PC pairing. Rather, synapsis-independent full-length intimate alignment of homologs implies a direct contribution of intrinsic pairing activity of non-PC regions of the chromosomes to the homolog recognition process. Such an ability of non-PC regions of chromosomes to mediate homologous associations was suggested both by previous observations of parallel chromosome axes in spread nuclei [35] and transient pairing in translocation heterozygotes of chromosome regions that ultimately become engaged in heterologous synapsis [20], [54] and by observation in the current analysis of chromosome pairs associated via non-PC regions. Moreover, our ability to visualize chromosome territories provides an opportunity to discover factors that mediate SC-independent full lengthwise alignment and to investigate their potential contributions to presynaptic alignment of homologs during wild-type meiosis. We recently identified a meiotic mutant that is substantially impaired for synapsis-independent full lengthwise alignment but retains proficiency for PC pairing activity (Dombecki et al, submitted), indicating that the process is under separate genetic control.

We integrate our observations from chromosome painting of wild-type, *chk-2*, *him-3* and *syp-1* mutants as well as *him-8*/PC-defective germ cells with data from previous studies to develop a possible model for homolog pairing during *C. elegans* meiosis. Specifically, we propose that synapsis-independent elongation of chromosome territories and a capacity for full-length alignment collaborate with other functions of PCs/PC binding proteins to bring about successful sorting of meiotic chromosomes into homologous pairs. According to this model, cytoskeletal driven chromosome movement facilitates bringing prospective pairing partners into proximity at defined sites (*i.e.* the PCs), whereas elongation and restructuring of chromosome territories enables rapid lengthwise juxtaposition of chromosome segments, and potentially of entire chromosomes. This lengthwise juxtaposition of homologs would facilitate assessment of suitability of potential pairing partners. Indeed, similarities in structure between homologous territories may contribute to homolog recognition *per se*. Further, the capacity for synapsis-independent full-length alignment and the relatively high stability of this configuration suggests the formation of presynaptic interactions between homologs that can resist tension exerted by cytoskeletal forces pulling the PCs of homologous chromosomes in opposite directions. Such forces have been postulated to play a role in a proposed checkpoint-like mechanism that functions to license SC assembly in response to homology verification [25], [27]. Although our analysis does not prove that elongation of chromosome territories directly facilitates the alignment process, this model helps to reconcile how highly localized chromosomal sites can serve to promote utilization of information about chromosome identity that is distributed along the length of a chromosome: by constraining an extended chromosome territory at a single point, the problem of aligning homologs in register is considerably simplified.

It is intriguing that, at least for the *X* chromosome, multiple distinct functions that promote homolog pairing and synapsis are coordinately dependent on a specific Zn-finger DNA binding protein and its distribution on the chromosome. We speculate that this feature may have been instrumental in the emergence in the nematode of a recombination–independent mechanism for achieving homologous synapsis. Consolidation of these functions in a single protein may have permitted evolution of a robust alternative mechanism for homology verification, thereby reducing reliance on the ancestral recombination-based strategy.

## Materials and Methods

### Genetics

All *C. elegans* strains were cultured at 20°C following standard conditions [55]. The following mutants and chromosome rearrangements as well as the wild type strain Bristol N2 were used: Chromosome *IV*: *him-8(e1489)*, *him-8(me4)*, *him-3(gk149)*, Chromosome *V*: *syp-1(me17)*, *chk-2*(*me64*), and the *X* chromosome: *meDf2(X)*, *mnDp66(I:X)*.

### Chromosome paint

Chromosome painting was done using a protocol for FISH described in [31] with modifications as briefly described in the following:

#### Probe preparation

The YAC compositions of painting probes used in this study are listed in Table S1, except for a partial *X* chromosome paint probe used for the *meDf2* analysis, which contains only groups 4–19 of the *X*-chromosome YACs. YAC DNA was prepared using the CHEF-DR II pulse-field electrophoresis system (Bio-Rad) following manufacturer's instructions, with agarose gels made of SeaKem GTG agarose (Cambrex). Agarose blocks containing YAC bands were excised from gels, digested by GELase (EPICENTRE Biotechnologies), and used as templates for amplification using the Illustra GenomiPhi V2 DNA Amplification Kit (GE health care). At the amplification step, multiple YAC clones were combined and processed as groups containing YACs covering approximately 1 Mb chromosomal regions, as indicated in Table S1. Amplified YAC DNA was digested using a cocktail of restriction enzymes (AluI, HaeIII, MseI, MspI, RsaI, and Sau3AI; New England Biolabs), and purified by QIAquick reaction cleanup kit (QIAGEN). Labeling was done with ULYSIS DNA labeling kit (Invitrogen), using 2 µg of purified DNA per reaction. After labeling, labeled DNA was purified with Centri-Sep columns (Applied Biosystems), and eluted into 20 µl solution. Multiple groups of labeled DNA (1 µl of each group per slide) were further combined as indicated in Table S1 to generate a paint probe; this cocktail was dehydrated using a speed vacuum concentrator, then resuspended in water before applying to the sample.

#### Sample preparation

Dissected gonads were incubated with dissection buffer [31] containing 10% Tween-20 for 10 minutes at room temperature, then fixed with 1% formaldehyde for 5 min. Fixed samples were frozen in liquid nitrogen and then processed in 95% ethanol at −20°C for 10 minutes. After washing in 2X SSCT (2X SSC containing 0.5% Tween-20), the concentration of formamide was increased to 50% in a stepwise manner. After pre-hybridization in 2XSSCT containing 50% formamide at 37°C for two hours, hybridization solution [31] containing a paint probe was added to the sample and slides were processed using a flat bed thermal cycler (OmniSlide, Thermo Fisher). Heat denaturation and subsequent hybridization were done at 77°C for 10 minutes and at 37°C over night, respectively. Slides were washed twice in 2XSSCT containing 50% formamide at 37°C for 30 minutes, followed by gradual removal of formamide from 2XSSCT. Slides were then incubated with 2XSSCT containing DAPI for 15 minutes, washed in 2XSSCT, and mounted with SlowFade Gold mounting medium (Invitrogen).

#### Imaging

All images were collected as 5-channel optical sections in increments of 0.1 µm, using a confocal microscope SP2 (Leica Microsystems). For Figure 1, Figure 2, Figure 3, Figure 4, Figure 5, and Figure 6, volume renderings were generated using the Volocity 4 Visualization software (PerkinElmer). For Figure 8A and Figure 9A, maximum-intensity Z-projections were generated with Priism/IVE software [56].

### Image analysis of chromosome paint

To evaluate the spatial distribution (Figure 2) alignment state (Figure 3, Figure 4, Figure 5, Figure 6, Figure 8, and Figure 9) and morphology (Figure 5, Figure 6, Figure 7, Figure 8, and Figure 9) of chromosome territories, we generated 3D volume renderings of nuclei from the relevant zones within the germ line using the Volocity 4 program. Each nucleus was examined individually by rotating the nucleus to allow assessment of the shapes of and spatial relationships between specified chromosome territories. All nuclei in the relevant zone that were well separated from neighboring nuclei were included in the analyses.

For quantitative analysis of alignment states in the *syp-1* mutant, zones were defined as follows. The gonad was first subdivided to two parts: pre-meiotic and meiotic (beginning at the transition zone). The total number of rows of nuclei (N) in the meiotic zone was divided by 5 and the quotient was rounded to the nearest integer (n); the width of zones 2–5 was set to n rows of nuclei, whereas the width of zone 6 was set to N-4n (usually a little smaller than n) rows. Data from 5 gonads were pooled.

For quantitation of “painted chromosomal segments” (Figure 6, Figure 7, Figure 8, Figure 9), segments were defined as objects that were distinctively segmented from other parts of territories in 3D renderings, *i.e.*, peaks of high signal intensity separated by gaps or regions of reduced signal intensity and/or spatially resolved signals from fluorophores painting different regions of the chromosome. For these analyses of the wild type (and the corresponding quantitation of alignment), nuclei were score in zones defined as follows: “pre-meiotic”, comprising nuclei within the first 10 rows from the distal tip; “transition zone”, which included all nuclei within the transition zone; “pachytene”, comprising nuclei in a 15–20 row zone beginning 5 rows from the proximal end of the transition zone. The length of the transition zone for the analyses presented in Figure 8 and Figure 9 was defined based on the status of alignment of chromosome *I* (see Results). The lengths of the transition zones defined in this manner were very similar among the wild type and mutants analyzed (Figure S1): 11±1, 8.6±2, 10.6±1.1 and 8±1 (mean ± SD) rows of nuclei for the wild type, *him-8(e1489)*, *him-8(me4)* and *mnDp66*; m*eDf2* respectively.

Statistical analyses were performed using the InStat program (Graphpad). A two-tailed Chi-square test was used for analysis of the data presented in Figure 2. A two-tailed Mann-Whitney test was used to analyze the “painted chromosome segment” data presented in Figure 6C, Figure 7B, Figure 8C and Figure 9C. A two-tailed Fisher exact test was used to analyze the data presented in Figure 8D and Figure 9D.

### Immunofluorescence

Immunofluorescence was performed essentially as described in [31], [57]. The following primary antibodies (dilutions) were used: guinea pig anti HIM-8 (1∶500) [21]; chicken anti HTP-3 (1∶250) [20]); rabbit anti SYP-1 (1∶250) [19]; mouse monoclonal anti H3K9me2 (1∶400) (Abcam). Alexafluor 488, 555, or 647-conjugated secondary antibodies were used at a dilution of 1∶500 (Invitrogen). Images were collected as 0.1 µm optical sections using the DeltaVision microscopy system and deconvolved using SoftWoRx 4.0.0 software (Applied Precision); we note that HIM-8 speckles were readily visible prior to deconvolution. Registration was corrected for chromatic shift and images were rendered using Volocity 5.5 software (PerkinElmer).

## Supporting Information

Figure S1 Spatial extents of transition zones as defined for analysis of *X* territory elongation. Maximum intensity projections of regions of DAPI-stained gonads encompassing the transition zone, from wild type, *him-8(e1489)*, *him-8(me4)* and *mnDp66; meDf2* worms; images shown were generated from confocal images used for the chromosome elongation analysis. The distal end of each gonad is towards the left. The region between the two dashed lines in each panel corresponds to the transition zone as operationally defined by the criteria described in the text. The length of the transition zone was similar among these strains, ruling out the possibility that the observed differences in chromosome elongation resulted from difference in the lengths of the zone of nuclei that were scored. Scale bar; 5 µm.
(TIF)

Figure S2 Illustration of multiple painted chromosome segments without a high degree of territory extension in an *X*-PC deletion homozygote. Three-dimensional rendered paint images of *X* chromosomes in a wild type pachytene nucleus (left) and a pachytene nucleus from a worm homozygous for *meDf2*, which is deleted for the *X*-PC(right). The left half of the *X* chromosome is painted by Alexa-594 (red) and the right half is painted by Alexa-647 (blue). DAPI is white (top). For each nucleus depicted, in the bottom panels, visually discernable painted chromosomal segments are marked with dots, which are connected by a line tracing the path of the chromosome territory. In the *meDf2* nucleus, both *X* chromosomes have 4 discernable painted segments. However, while one of the *X* chromosome territories has a more extended linear organization, the other *X* chromosome (arrow) has a more compact territory that reflects a folded configuration of the chromosome. Scale is shown by the square grid in the background of each panel, with 3.8 µm as the length of each side of the unit square.
(TIF)

Table S1 Summary of YAC clones used to generate the indicated paint probes. LE: The distance from the left end of the chromosome to the left end of the clone (Mb). RE: The distance from the left end of the chromosome to the right end of the clone (Mb). Label: The fluorophore used to label the indicated YAC or group of YACs.
(PDF)

Video S1 Quantitation of painted segments for chromosome *I*. A movie of a rotating three-dimensional rendered paint image of the nucleus shown in the bottom panel of Figure 6A, depicting a partially aligned chromosome *I* pair. The left half of chromosome *I* is painted by Alexa-488 (green) and the right half is painted by Alexa-532 (yellow). Individual segments counted in the assay are marked by purple cubes, which are connected by solid lines tracing the paths of the chromosomes.
(MOV)

Video S2 Quantitation of painted segments for the *X* chromosome. A movie of a rotating three-dimensional rendered paint image of the nucleus shown in the bottom panel of Figure 6B, depicting an unaligned pair of *X* chromosomes. The left half of the *X* chromosome is painted by Alexa-594 (red) and the right half is painted by Alexa-647 (blue). DAPI is shown in white. Individual segments counted in the assay are marked by green or orange cubes, which are connected by solid lines tracing the paths of the chromosomes.
(MOV)
