# Supplementary figures and images for: Chromosome Painting Reveals Asynaptic Full Alignment of Homologs and HIM-8–Dependent Remodeling of X Chromosome Territories during Caenorhabditis elegans Meiosis
Source: PLoS Genet. 2011 Aug 18;7(8):e1002231. doi: 10.1371/journal.pgen.1002231 (PMC3158051; doi:10.1371/journal.pgen.1002231)

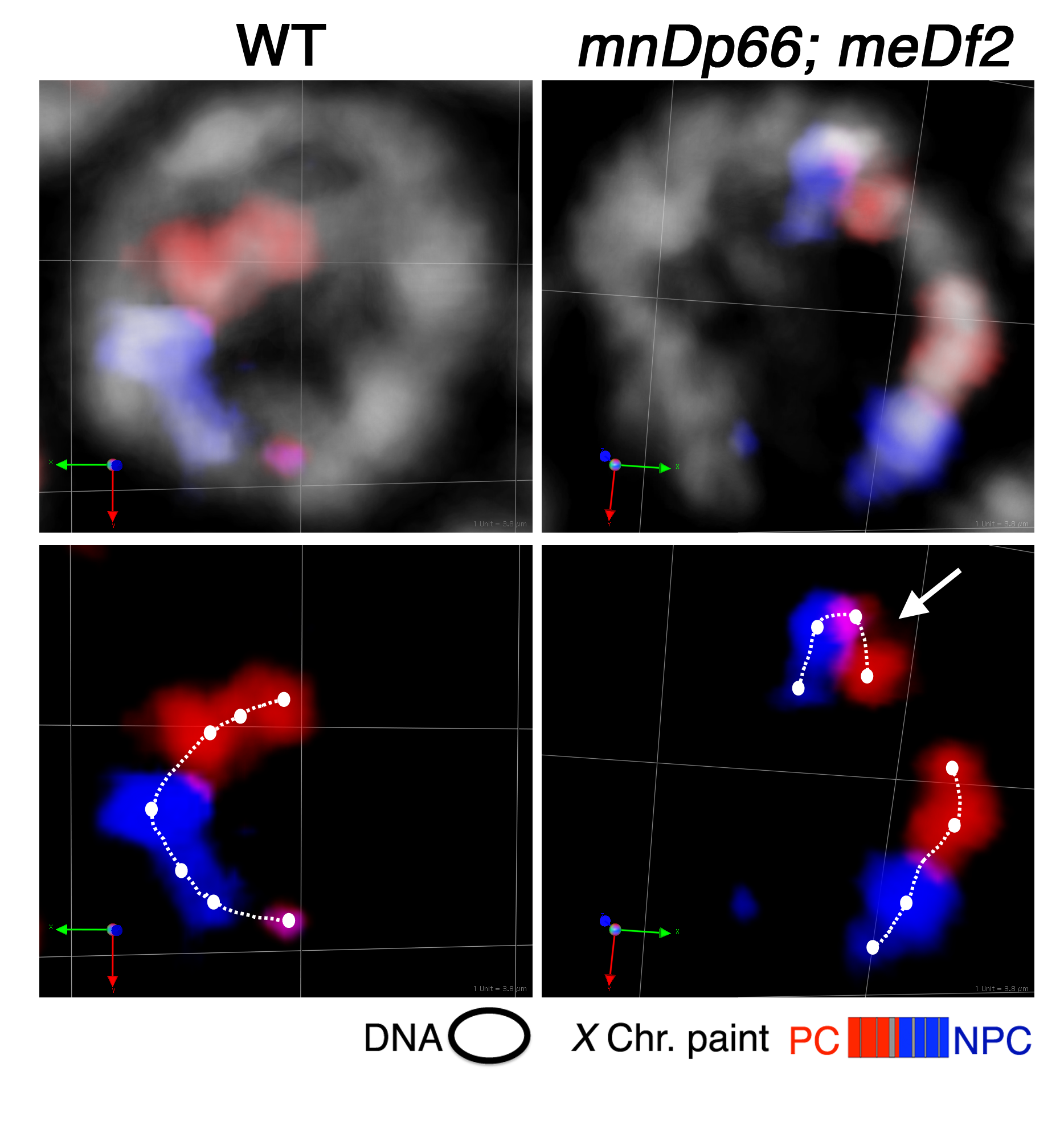

Supplement: Figure S2 — Illustration of multiple painted chromosome segments without a high degree of territory extension in an X-PC deletion homozygote. Three-dimensional rendered paint images of X chromosomes in a wild type pachytene nucleus (left) and a pachytene nucleus from a worm homozygous for meDf2, which is deleted for the X-PC(right). The left half of the X chromosome is painted by Alexa-594 (red) and the right half is painted by Alexa-647 (blue). DAPI is white (top). For each nucleus depicted, in the bottom panels, visually discernable painted chromosomal segments are marked with dots, which are connected by a line tracing the path of the chromosome territory. In the meDf2 nucleus, both X chromosomes have 4 discernable painted segments. However, while one of the X chromosome territories has a more extended linear organization, the other X chromosome (arrow) has a more compact territory that reflects a folded configuration of the chromosome. Scale is shown by the square grid in the background of each panel, with 3.8 µm as the length of each side of the unit square. (TIF) [file pgen.1002231.s002.tif]
