# Supplementary material for: Chromosome Painting Reveals Asynaptic Full Alignment of Homologs and HIM-8–Dependent Remodeling of X Chromosome Territories during Caenorhabditis elegans Meiosis
Source: PLoS Genet. 2011 Aug 18;7(8):e1002231. doi: 10.1371/journal.pgen.1002231 (PMC3158051; doi:10.1371/journal.pgen.1002231)
Supplement: Table S1 — Summary of YAC clones used to generate the indicated paint probes. LE: The distance from the left end of the chromosome to the left end of the clone (Mb). RE: The distance from the left end of the chromosome to the right end of the clone (Mb). Label: The fluorophore used to label the indicated YAC or group of YACs. (PDF) [file pgen.1002231.s003.pdf]

Chromosome II (3 color)

Group	Clone	LE	RE	Label
**1**	Y43H11	0.2	0.4	**Alexa-488**
	Y54A7	0.4	0.6	
	Y39F10	0.6	0.9	
**2**	Y42D11	0.9	1.3	**Alexa-488**
	Y25C1	3.0	3.2	
	Y46D2	3.3	3.6	
**3**	Y8A9	3.9	4.2	**Alexa-488**
	Y41E4	4.1	4.4	
	Y54C5	4.4	4.6	
**4**	Y52E8	4.6	4.9	**Alexa-488**
	Y49D8	4.9	5.2	
**6**	Y54E1	5.1	5.7	**Alexa-546**
	Y3D6	5.7	5.8	
	Y65C9	5.8	6.0	
**7**	Y97C10	6.0	6.7	**Alexa-546**
	Y76G10	6.7	7.0	
	Y47A12	7.0	7.2	
**8**	Y39G9	7.2	7.5	**Alexa-546**
	Y103A9	7.3	8.3	
**9**	Y97G3	8.4	8.9	**Alexa-546**
	Y45F4	8.8	9.2	
**10**	Y9C2	9.3	9.6	**Alexa-546**
	Y97H8	9.5	9.8	
	Y13E5	9.8	9.9	
**11**	Y97D12	9.9	10.3	**Alexa-546**
	Y51F9	10.2	10.5	
	Y105B8	10.9	11.4	
**12**	Y54E11	11.4	11.8	**Alexa-546**
	Y102G4	11.7	12.2	
	Y65C11	12.2	12.3	
**13**	Y81G3	13.2	13.5	**Alexa-647**
	Y48E1	13.6	13.8	
	Y54G9	13.7	13.9	
	Y51H1	13.9	14.1	
**14**	Y108H6	14.0	14.8	**Alexa-647**
	Y14A1	14.8	14.9	
	Y53F4	14.9	15.2	
**15**	Y43H11	0.2	0.4	**Alexa-647**
**16**	Y53F4	14.9	15.2	**Alexa-488**

Chromosome I (2 color)

Group	Clone	LE	RE	Label
**1**	Y73F10	0.0	0.2	**Alexa-488**
	Y50C1	0.2	0.5	
	Y65B4	0.4	0.6	
**2**	Y18H1	0.6	0.7	**Alexa-488**
	Y73A3	0.7	0.9	
	Y34D9	1.0	1.2	
**3**	Y48G8	1.2	1.3	**Alexa-488**
	Y52D1	1.3	1.5	
	Y71G12	1.5	1.7	
**4**	Y115A10	2.3	2.5	**Alexa-488**
	Y44E3	2.6	2.7	
	Y74A12	2.7	3.0	
**5**	Y74A11	3.0	3.5	**Alexa-488**
	Y39E12	3.5	3.6	
	Y40G6	3.8	4.0	
**6**	Y110A7	3.9	4.7	**Alexa-488**
**7**	Y70C6	5.1	5.3	**Alexa-488**
	Y46D1	5.3	5.5	
	Y54B12	5.4	5.5	
**8**	Y101C10	5.5	6.3	**Alexa-488**
	Y39A9	6.2	6.6	
**9**	Y53F1	6.6	6.9	**Alexa-488**
	Y97F9	6.8	7.6	
**10**	Y97D1	7.6	8.0	**Alexa-532**
	Y97E2	8.0	8.5	
**11**	Y43C3	8.5	8.9	**Alexa-532**
	Y43E2	8.9	9.0	
	Y49G9	9.0	9.2	
**12**	Y102E5	9.1	9.5	**Alexa-532**
	Y106G6	9.5	10.3	
**13**	Y71B8	10.3	10.4	**Alexa-532**
	Y19G12	10.5	10.7	
	Y37F4	10.7	10.8	
**14**	Y95D11	10.8	10.9	**Alexa-532**
	Y53A2	11.2	11.6	
	Y47H9	11.5	11.9	
**15**	Y47H10	12.0	12.4	**Alexa-532**
	Y45E10	12.2	12.5	
	Y91F4	12.5	12.7	
**16**	Y50A7	12.7	13.1	**Alexa-532**
	Y43D10	13.0	13.5	
**17**	Y40B1	13.3	13.6	**Alexa-532**
	Y63D3	14.1	14.2	

Chromosome II (2 color)

Group	Clone	LE	RE	Label
**1**	Y43H11	0.2	0.4	**Alexa-594**
	Y54A7	0.4	0.6	
	Y39F10	0.6	0.9	
**2**	Y42D11	0.9	1.3	**Alexa-594**
	Y25C1	3.0	3.2	
	Y46D2	3.3	3.6	
**3**	Y8A9	3.9	4.2	**Alexa-594**
	Y41E4	4.1	4.4	
	Y54C5	4.4	4.6	
**4**	Y52E8	4.6	4.9	**Alexa-594**
	Y49D8	4.9	5.2	
**6**	Y54E1	5.1	5.7	**Alexa-594**
	Y3D6	5.7	5.8	
	Y65C9	5.8	6.0	
**7**	Y97C10	6.0	6.7	**Alexa-594**
	Y76G10	6.7	7.0	
	Y47A12	7.0	7.2	
**8**	Y39G9	7.2	7.5	**Alexa-594**
	Y103A9	7.3	8.3	
**9**	Y97G3	8.4	8.9	**Alexa-647**
	Y45F4	8.8	9.2	
**10**	Y9C2	9.3	9.6	**Alexa-647**
	Y97H8	9.5	9.8	
	Y13E5	9.8	9.9	
**11**	Y97D12	9.9	10.3	**Alexa-647**
	Y51F9	10.2	10.5	
	Y105B8	10.9	11.4	
**12**	Y54E11	11.4	11.8	**Alexa-647**
	Y102G4	11.7	12.2	
	Y65C11	12.2	12.3	
**13**	Y81G3	13.2	13.5	**Alexa-647**
	Y48E1	13.6	13.8	
	Y54G9	13.7	13.9	
	Y51H1	13.9	14.1	
**14**	Y108H6	14.0	14.8	**Alexa-647**
	Y14A1	14.8	14.9	
	Y53F4	14.9	15.2	

***X* Chromosome (2 color)**

Group	Clone	LE	RE	Label
**1**	Y35H6	0.1	0.2	**Alexa-594**
	Y47C4	0.3	0.4	
	Y51E2	0.5	1.0	
**2**	Y102A12	1.1	1.8	**Alexa-594**
**3**	Y105G12	1.4	2.1	**Alexa-594**
**4**	Y97B8	2.4	2.9	**Alexa-594**
**5**	Y76F7	2.9	3.1	**Alexa-594**
	Y40H5	3.2	3.4	
	Y43D5	3.4	3.7	
**6**	Y18F11	3.8	4.0	**Alexa-594**
	Y39H11	4.0	4.2	
	Y52A10	4.2	4.5	
**7**	Y52C2	4.6	5.0	**Alexa-594**
	Y37D3	5.3	5.5	
**8**	Y53D7	5.6	5.9	**Alexa-594**
	Y23B4	5.8	6.0	
	Y72A10	6.0	6.3	
**9**	Y43B9	6.4	6.6	**Alexa-594**
	Y40F6	6.6	6.8	
	Y97D3	7.0	7.4	
**10**	Y18C11	7.4	7.7	**Alexa-594**
	Y50C2	8.4	9.0	
**11**	Y70G9	9.1	9.4	**Alexa-647**
	Y44D2	9.4	9.7	
**12**	Y102D2	9.7	10.2	**Alexa-647**
	Y97D4	10.0	10.4	
**13**	Y97D9	10.5	11.0	**Alexa-647**
	Y108G6	10.9	11.4	
**14**	Y75H1	11.9	12.0	**Alexa-647**
	Y39F3	12.2	12.7	
	Y40A3	12.7	12.9	
**15**	Y45E9	12.9	13.3	**Alexa-647**
	Y50B10	13.1	13.6	
**16**	Y31A8	14.0	14.1	**Alexa-647**
	Y52C11	14.1	14.6	
	Y42D5	14.5	14.9	
**17**	Y53A6	14.9	15.5	**Alexa-647**
	Y7A5	15.6	15.8	
**18**	Y46E1	15.7	16.0	**Alexa-647**
	Y50B3	16.0	16.1	
	Y25B5	16.5	16.7	
**19**	Y43F3	16.7	16.9	**Alexa-647**
	Y52F1	16.9	17.2	
	Y68A3	18	18	
